# Endothelial PDGF-CC regulates angiogenesis-dependent thermogenesis in beige fat

**DOI:** 10.1038/ncomms12152

**Published:** 2016-08-05

**Authors:** Takahiro Seki, Kayoko Hosaka, Sharon Lim, Carina Fischer, Jennifer Honek, Yunlong Yang, Patrik Andersson, Masaki Nakamura, Erik Näslund, Seppo Ylä-Herttuala, Meili Sun, Hideki Iwamoto, Xuri Li, Yizhi Liu, Nilesh J. Samani, Yihai Cao

**Affiliations:** 1 Department of Microbiology, Tumor and Cell Biology, Karolinska Institute, Stockholm 171 77, Sweden; 2 Department of Clinical Sciences, Danderyd Hospital, Karolinska Institute, Stockholm 182 88, Sweden; 3 Department of Molecular Medicine, A.I. Virtanen Institute, Molecular Sciences University of Eastern Finland, Kuopio 70211, Finland; 4 State Key Laboratory of Ophthalmology, Zhongshan Ophthalmic Center, Sun Yat-Sen University, Guangzhou 510060, China; 5 Department of Cardiovascular Sciences, University of Leicester and NIHR Leicester Cardiovascular Biomedical Research Unit, Glenfield Hospital, Leicester LE3 9QP, UK

## Abstract

Cold- and β3-adrenoceptor agonist-induced sympathetic activation leads to angiogenesis and UCP1-dependent thermogenesis in mouse brown and white adipose tissues. Here we show that endothelial production of PDGF-CC during white adipose tissue (WAT) angiogenesis regulates WAT browning. We find that genetic deletion of endothelial VEGFR2, knockout of the *Pdgf-c* gene or pharmacological blockade of PDGFR-α impair the WAT-beige transition. We further show that PDGF-CC stimulation upregulates UCP1 expression and acquisition of a beige phenotype in differentiated mouse WAT-PDGFR-α^+^ progenitor cells, as well as in human WAT-PDGFR-α^+^ adipocytes, supporting the physiological relevance of our findings. Our data reveal a paracrine mechanism by which angiogenic endothelial cells modulate adipocyte metabolism, which may provide new targets for the treatment of obesity and related metabolic diseases.

## Abstract (web summary)

Cold-induced activation of thermogenesis in white adipose tissue (WAT), or ‘beiging', is associated with WAT angiogenesis. Here the authors show that PDGF-CC is secreted from endothelial cells in the context of WAT angiogenesis and its paracrine action on adipocytes contributes to cold-induced beiging.

## Graphical abstract

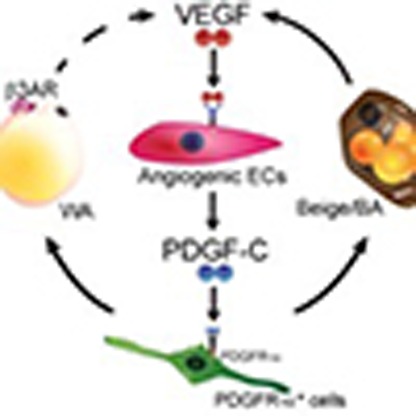

Adipose tissues, especially brown adipose tissue (BAT) are highly vascularized and the adipose vasculature exhibits plasticity features depending on the metabolic status of adipocytes^1^,^2^. In fact, adipose tissues relentlessly experience expansion and shrinkage throughout adulthood and the adipose plasticity demands microvessel growth or regression to cope with adipose functions of energy deposition or expenditure. The adipose vasculature may have multifarious functions^1^,^2^, including supply of nutrients and oxygen to adipocytes and maintaining their optimal functions and survival; removal of metabolic products from adipose tissues; conductance of heat to the rest of the body; transportation of lipid molecules for energy deposition or expenditure; providing circulating cells to modulate the cellular composition in the adipose microenvironment; bidirectional transportation of circulating hormones, growth factors, cytokines and adipokines to modulate functions of adipose and non-adipose tissues; and the vessel wall as a reservoir of stem cells that potentially differentiate into preadipocytes and adipocytes. Recent studies support the fact that the Zfp243^+^ committed preadipocytes are located to adipose endothelial and perivascular cells and they can differentiate into both white and brown adipocytes^3^,^4^; preparation of the initial adipose niche formation during embryogenesis; maintenance of adipose tissue architectures; modulation of the adipose microenvironment such as tissue hypoxia that regulates gene expression, cell differentiation and infiltration; and possible modulation of adipocyte functions via paracrine regulatory mechanisms. Although adipocyte-derived factors and cytokines in regulation of angiogenesis are relatively well studied, the role of ECs in modulation of adipocyte growth, differentiation and function remains less understood.

Vascular ECs and adipocytes are two principal cellular components in the adipose microenvironment, and they intimately crosstalk to each other by producing various soluble and cell surface-bound factors^1^. WAT and BAT adipocytes produce various angiogenic factors, cytokines and adipokines that regulate angiogenesis, vascular survival, vascular remodelling and blood perfusion. For example, vascular endothelial cell growth factor (VEGF) is one of the key angiogenic factors in angiogenic adipose tissues^5^,^6^,^7^,^8^,^9^. VEGF binds to VEGFR1 and VEGFR2, two tyrosine kinase receptors, primarily expressed on ECs^10^,^11^,^12^. Abundant evidence shows that VEGFR2, but not VEGFR1, transduces VEGF-induced angiogenic, permeability and other vascular functions, whereas VEGFR1 may act as a decoy receptor^10^,^11^,^12^,^13^,^14^. Members in the PDGF family share structural and functional similarities, and their biological functions are transduced through PDGFR-α and PDGFR-β distributed on various cell types^15^. In addition to the formation of their homodimers, PDGFR-α and PDGFR-β can also form heterodimers in cells that co-express these two receptors. PDGF-CC is able to bind to PDGFR-α homodimers and PDGFR-α/PDGFR-β heterodimers and induces angiogenesis and vascular homoeostasis in *in vivo* animal models^16^,^17^,^18^,^19^.

Recent studies also show that cold-induced sympathetic activation markedly augments adipose angiogenesis during browning of subcutaneous WAT and VEGF is the key angiogenic mediator in this experimental setting^20^,^21^,^22^. Similar to cold exposure, adrenergic activation by β3-adrenergic agonist (CL316,243, termed CL throughout this article) is able to induce a similar browning beige phenotype and BAT activation^23^,^24^,^25^,^26^,^27^,^28^. Transition from WAT to browning beige adipose tissue involves transcriptional regulation of multiple BAT-associated gene products that execute BAT-like functions. For example, mitochondrial uncoupling protein1 (UCP1) is specifically upregulated under this condition and is required for non-shivering thermogenesis^29^,^30^,^31^,^32^. In addition, several transcription factors and lipid metabolism-related enzymes including peroxisome proliferator-activated receptor gamma coactivator-1 alpha (PGC-1α), PRD1-BF1-RIZ1 homologous domain containing 16 (PRDM16), cell death activator CIDE-A (CIDEA), and cytochrome c oxidase, subunit 8B pseudo gene (COX8B) are expressed in browning adipocytes and interscapular BAT adipocytes^23^,^25^. The adipose PDGFR-α-positive cell population shows distinctive morphology and defines as bi-potential adipocyte progenitors that can differentiate into BAT and WAT adipocytes *in vitro* and *in vivo*, suggesting that PDGFR-α-triggered signalling determines preadipocyte fate and function. However, cellular source and ligand identity that activates the PDGFR-α signalling system in relation to modulation of adipocyte differentiation remains uncharacterized. Particularly, the PDGF-PDGFR-α signalling in relation to the adipose vasculature in modulation of adipocyte function is unknown.

In the present study, we employed several genetic knockout mouse models in combination with specific pharmacological blocking and neutralizing agents to demonstrate the existence of an endothelial paracrine mechanism that controls adipocyte functions. Both loss-of-function and gain-of-function studies showed that the VEGF-VEGFR2 signalling controls endothelial production of PDGF-CC that mediates the paracrine communication with adipocytes. Genetic deletion or treatment with specific inhibitors of each of these components virtually impairs the β3-adrenergic or cold-induced beige phenotype. These data are physiologically relevant since inhibition of the PDGFR-α-triggered signalling significantly attenuates cold-induced non-shivering thermogenesis. Our findings suggest that intervention of PDGF signalling pathways may have therapeutic implications for treatment of obesity and the related metabolic diseases.

## Results

### Induction of adipose angiogenesis by adrenergic activation

**Figure 1:**
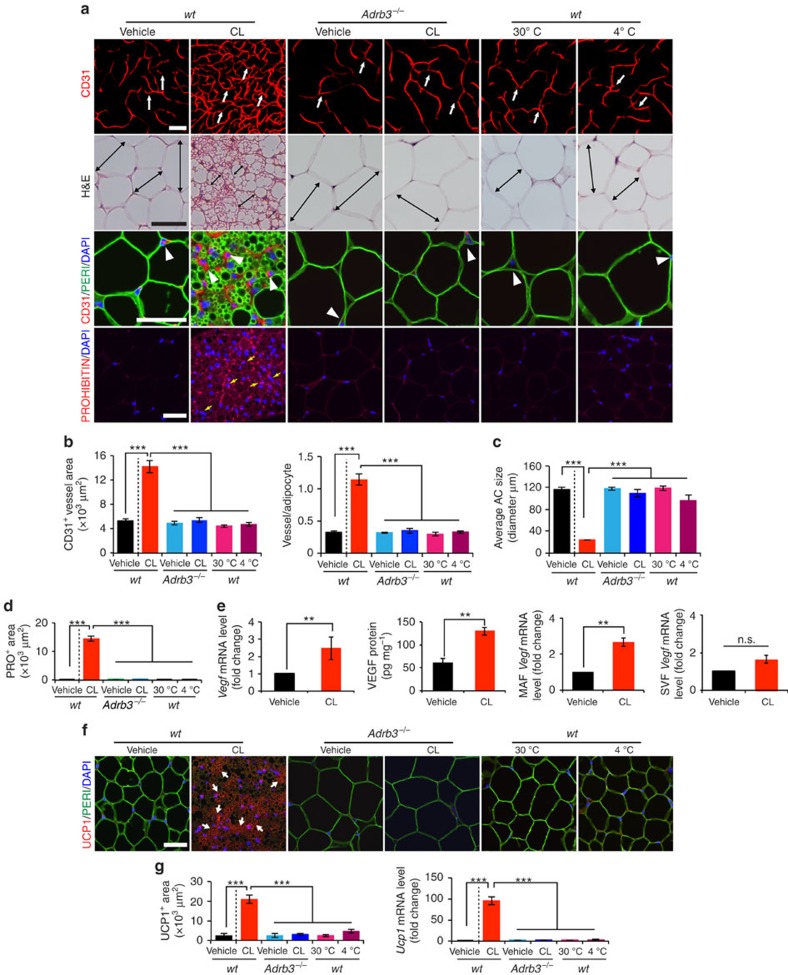
β3-adrenoceptor-dependent activation of adipose angiogenesis and the beige phenotype in visceral adipose tissue. (**a**) Microvessels (CD31^+^ red), adipocyte morphology (H&E; Perilipin^+^ green), and mitochondrial staining (Prohibitin^+^ red) of buffer-treated control (vehicle) and CL-treated gWAT in wild-type (wt) and *Adrb3*^*−/−*^ mice. White arrows and arrowheads point to microvessels and double-arrowed bars indicate adipocyte diameter. Yellow arrows point to prohibitin-positive signals. Cold (4 °C)- and thermoneutral temperature (30 °C)-exposed gWAT served as controls. *n*=5 mice for each group. (**b**) Quantification of microvessel density in various agent- or temperature-treated groups (*n*=10 random fields; *n*=5 mice for each group). (**c**) Quantification of average adipocyte size (>30 adipocytes per field; *n*=10 random fields; *n*=5 mice for each group). (**d**) Quantification of prohibitin-positive signals in various agents- or temperature-treated groups (*n*=10 random fields; *n*=5 mice for each group). (**e**) *Vegf* mRNA and protein expression in vehicle- and CL-treated gWAT samples. Total adipose tissue, MAF and SVF were used as starting materials (*n*=5 samples for each group). (**f**) UCP1 staining of gWAT in various agents- or temperature-treated wt and *Adrb3*^*−/−*^ mice. (*n*=5 samples for each group). Arrowheads point to UCP1-positive signals. Red=UCP1; green=perilipin; blue=DAPI. (**g**) Quantification of UCP1-positive staining signals (left, *n*=10 random fields) and mRNA (right, *n*=6–8 samples for each group). All scale bars, 50 μm. **P<0.01; ***P<0.001 by two-sided unpaired *t*-test. Data presented as mean±s.e.m. DAPI, 4',6-diamidino-2-phenylindole. n.s., not significant.

To investigate the role of angiogenesis in modulation of adipocyte functions, we chose a physiologically relevant and non-invasive *in vivo* model for our study. Administration of a β3-adrenergic agonist CL to 7–8 weeks old C57Bl/6 mice resulted in a robust angiogenic phenotype in gonadal WAT (gWAT) (Fig. 1a,b). Time course analysis showed that the CL-triggered angiogenesis occurred 2 days after CL treatment and an approximate threefold increase of CD31^+^ microvessel density was detected at day 10 after CL treatment (Supplementary Fig. 1a,b). Similar to gWAT, the CL-induced robust angiogenic phenotype also existed in other WAT depots including subcutaneous inguinal WAT (iWAT) (Supplementary Fig. 2a,b). CL treatment also induced a prominent angiogenic phenotype in interscapular BAT (iBAT) (Supplementary Fig. 2c,d). CL had no direct effects on capillary EC proliferation (Supplementary Fig. 3a). Expectedly, deletion of β3-adrenergic receptor in mice (*Adrb3*^*−/−*^) largely prevented CL-induced angiogenesis in WAT and BAT (Fig. 1a,b and Supplementary Fig. 2). Similarly, cold acclimation also produced a comparable angiogenic response in iWAT and iBAT. However, cold exposure was unable to induce adipose significant angiogenesis in gWAT (Fig. 1a,b), suggesting that cold-induced sympathetic activation preferentially stimulates angiogenesis in subcutaneous WAT depots. Concomitant to adipose angiogenesis, CL treatment resulted in a beige adipocyte phenotype in gWAT (a browning phenotype, also called a beige or a brite phenotype; for convenience; we used ‘beige' throughout this article), exhibiting smaller adipocyte sizes, marked increase of mitochondrial contents, and high expression levels of UCP1 mRNA and protein (Fig. 1a,c,d,f,g and Supplementary Figs 1a,d−f and 2e). UCP1 upregulation was restricted to the adipocyte fraction but not in the vascular fraction of the CL-treated gWAT (Supplementary Fig. 1f). Cold exposure also induced a comparable beige phenotype in iWAT, but not in gWAT (Fig. 1a and Supplementary Fig. 2a,b,e).

We next analysed mRNA and protein levels of VEGF and found that CL induced substantial upregulation of VEGF in iWAT and gWAT (Fig. 1e and Supplementary Fig. 1g). Time course study showed that the maximal level of VEGF expression occurred at day 3 and gradually decreased afterwards (Supplementary Fig. 1c). Fractionation analysis showed that VEGF levels were significantly upregulated in the mature adipocyte fraction (MAF) in which white and beige adipocytes were not separated (Fig. 1e). Inversely, CL did not significantly alter VEGF expression level in the stromal vascular fraction (SVF) (Fig. 1e).

### UCP1-independent adipose angiogenesis

**Figure 2:**
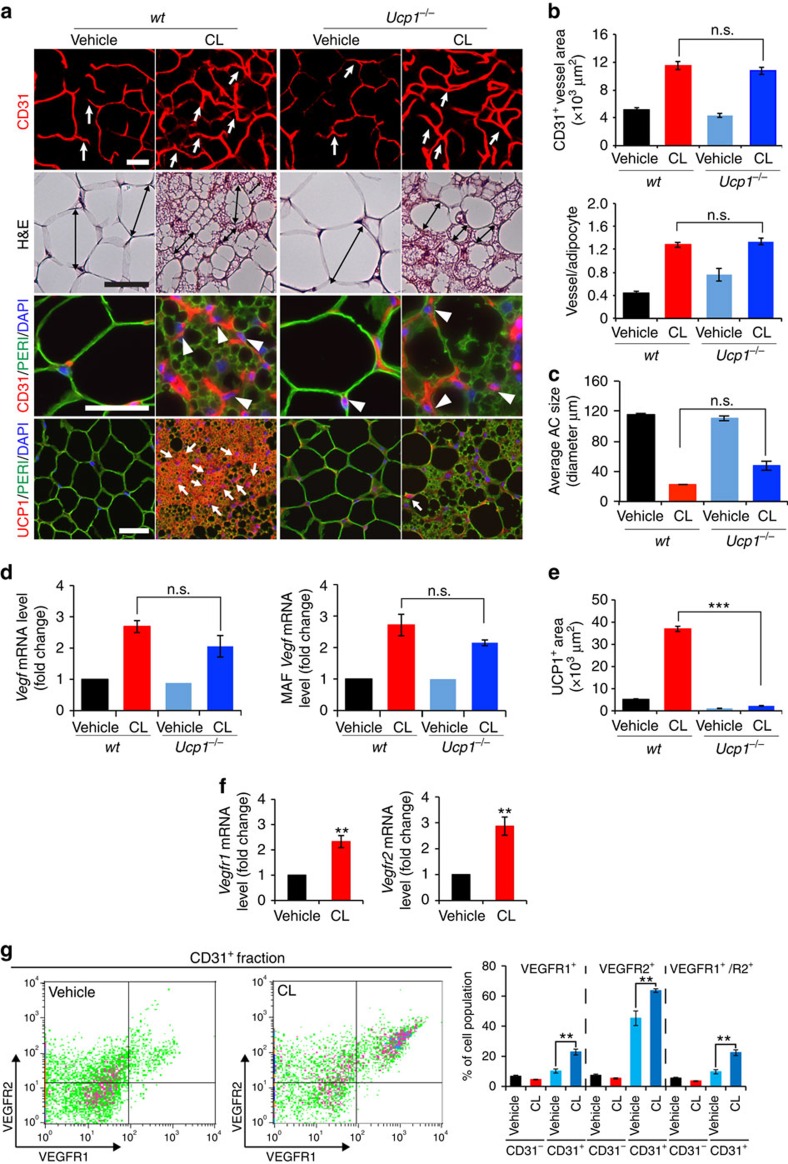
CL induces UCP1-independent adipose angiogenesis and upregulates VEGFR expression levels. (**a**) Microvessels (CD31^+^ red), adipocyte morphology (H&E; perilipin^+^ green), and UCP1 expression (UCP1^+^ red) of vehicle- and CL-gWAT in wt and *Ucp1*^*−/−*^ mice. White arrows and arrowheads in upper three panels point to microvessels. Arrows in the lowest row of panels indicate UCP1-positive signals. Double-arrowed bars mark adipocyte diameter. *n*=5 mice for each wt and *Ucp1*^*−/−*^ group. (**b**) Quantification of microvessel density in vehicle- and CL-treated wt and *Ucp1*^*−/−*^ mice (*n*=10 random fields; *n*=5 mice for each group). (**c**) Quantification of the average adipocyte size (>30 adipocytes/field; *n*=10 random fields; *n*=5 mice for each group). (**d**) *Vegf* mRNA expression levels in vehicle- and CL-treated total gWAT (left) and MAF (right) samples in wt and *Ucp1*^*−/−*^ mice (*n*=10 samples for each group). (**e**) Quantification of UCP1-positive signals (*n*=10 random fields; *n*=5 mice for each group). (**f**) *Vegfr1* and *Vegfr2* mRNA expression levels in vehicle- and CL-treated total gWAT samples in wt mice (*n*=10 samples for each group). (**g**) FACS analysis of VEGFR1 and VEGFR2 expression levels in CD31^+^ and CD31^−^ ECs isolated from vehicle- and CL-treated gWAT in wt mice (*n*=5 samples for each group). All scale bars, 50 μm. **P<0.01; ***P<0.001 by two-sided unpaired *t*-test. Data presented as mean±s.e.m. n.s., not significant.

One possible mechanism by which CL induced angiogenesis was that adrenergic stimulation led to activation of the UCP1-dependent thermogenic metabolism in adipose tissues, which subsequently induced angiogenesis. We therefore used UCP1-deficient (*Ucp1*^*−/−*^) mice to study adipose angiogenesis. CL-induced adrenergic activation in *Ucp1*^*−/−*^ mice did not prevent angiogenesis in gWAT, iWAT and iBAT (Fig. 2a,b and Supplementary Fig. 3b). Similarly, CL-induced beige phenotypic changes in gWAT were not altered in *Ucp1*^*−/−*^ mice relative to those seen in wild-type (wt) mice (Fig. 2a,c). UCP1-specific immunostaining confirmed that gWAT, iWAT and iBAT lacked UCP1 expression in *Ucp1*^*−/−*^ mice (Fig. 2a,e and Supplementary Fig. 3b). Consistent with the angiogenic phenotype, VEGF levels in total tissues and MAF were not altered in gWAT between *Ucp1*^*−/−*^ and wt mice (Fig. 2d). These findings demonstrate that CL-induced adipose angiogenesis occurs in the upstream of UCP1 and *via* an UCP1-independent mechanism.

We next analysed the effect of CL on expression levels of VEGF receptors (VEGFRs), and CL stimulation markedly augmented VEGFR1 and VEGFR2 expression levels in iWAT and gWAT relative to the vehicle-treated controls (Fig. 2f and Supplementary Fig. 3c). Fluorescence-activated cell sorting (FACS) analysis showed that VEGFR1^+^, VEGFR2^+^ and VEGFR1^+^/VEGFR2^+^ double-positive cell populations were markedly increased in the CD31^+^ fraction isolated from CL-treated gWAT relative to those in vehicle-treated control samples (Fig. 2g). In contrast, VEGFR1, VEGFR2 and VEGFR1/VEGFR2 expression levels were not increased in the CD31^−^ fraction (Fig. 2g). Compared with CD31^+^ cell fractions, CD31^−^ populations expressed very low levels of VEGFR1 and VEGFR2 (Fig. 2g). These findings show that adrenergic activation leads to upregulation of VEGFRs in the CD31^+^-positive fraction of adipose tissues. Thus, adrenergic activation-induced beige adipose tissues exhibit increased expression levels of VEGF and its receptors.

### VEGFR2 in beige phenotype and metabolism

**Figure 3:**
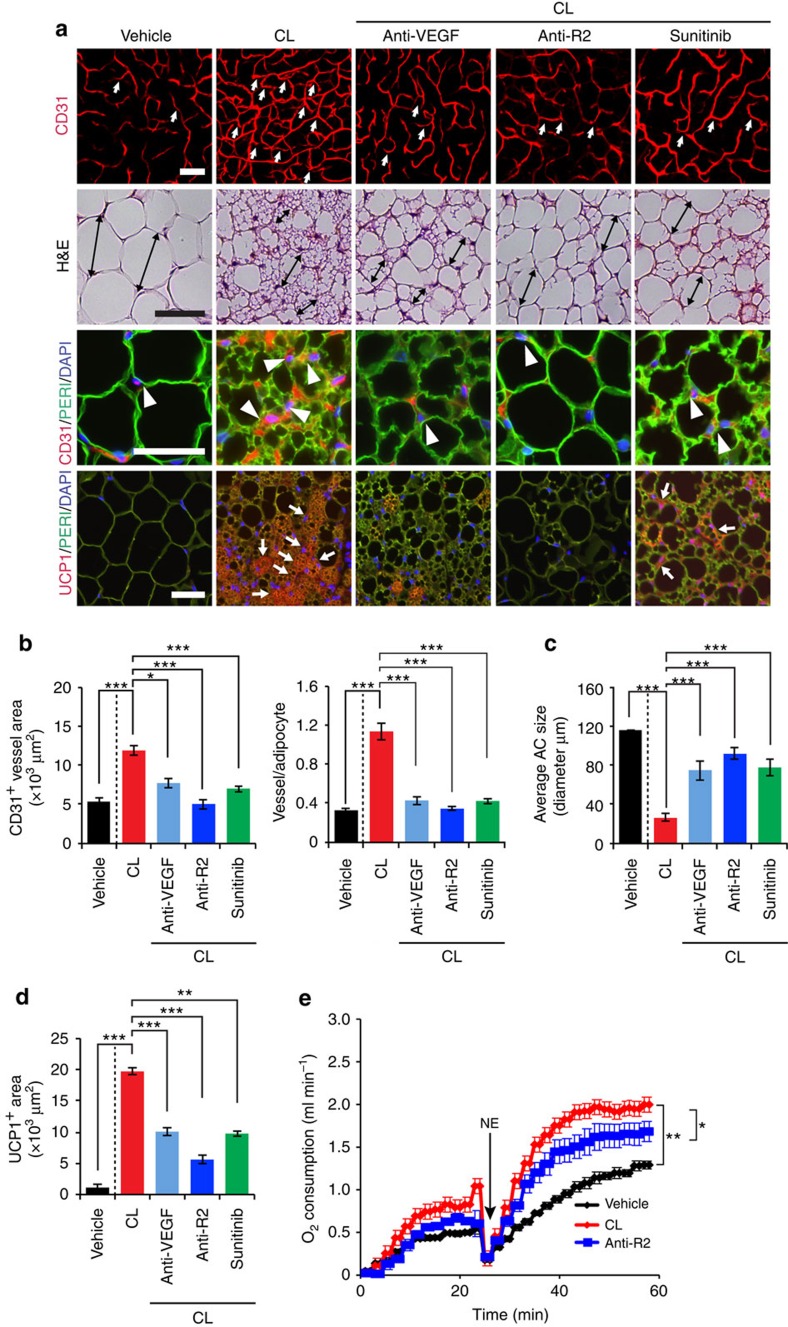
Effects of VEGF and VEGFR blockades on CL-induced adipose angiogenesis and beige phenotype. (**a**) Microvessels (CD31^+^ red), adipocyte morphology (H&E; perilipin^+^ green) and UCP1 expression (UCP1^+^ red) of CL-treated gWAT in mice that were treated with vehicle, VEGF-, and VEGFR2-specific neutralizing antibodies (Anti-VEGF and Anti-R2) and sunitinib. The vehicle- treated group served as a control. White arrows and arrowheads in upper three panels point to microvessels. Arrows in the lowest row of panels indicate UCP1-positive signals. Double-arrowed bars mark adipocyte diameter. Green=perilipin; blue=DAPI. *n*=8 for each vehicle, VEGF and VEGFR blockades group. (**b**) Quantification of microvessel density in vehicle- and CL-treated wt mice that received treatment with various VEGF blockades (*n*=10 random fields; *n*=8 mice for each group). (**c**) Quantification of average adipocyte size (>30 adipocytes/field; *n*=10 random fields; *n*=8 mice for each group). (**d**) Quantification of UCP1-positive signals (*n*=10 random fields; *n*=8 mice for each group). (**e**) Norepinephrine-stimulated non-shivering thermogenesis in VEGF/VEGFR blockade- and vehicle-treated mice that received CL or buffer (*n*=5 mice for each group). All scale bars, 50 μm. *P<0.05; **P<0.01; ***P<0.001 by two-sided unpaired *t*-test. Data presented as mean±s.e.m. NE, norepinephrine.

We then studied the VEGF-VEGFR signalling system in modulation of CL-induced adipose angiogenesis and adipocyte functions using a panel of VEGF- and VEGFR-specific neutralizing antibodies (VEGF and VEGFR2 blockade) and a VEGFR tyrosine kinase inhibitor (TKI), sunitinib^33^,^34^. An anti-VEGFR2, specific neutralizing antibody significantly prevented CL-induced angiogenesis in gWAT (Fig. 3a,b). Treatment with sunitinib produced a similar inhibitory effect on CL-induced angiogenesis in gWAT. To define VEGFR2 ligand, an anti-mouse VEGF-specific neutralizing antibody^35^ was used for treatment, which produced marked suppression of adipose angiogenesis. These findings show that the VEGF-VEGFR2 signalling pathway is responsible for adrenergic activation-induced angiogenesis in beige tissues.

To study the impact of VEGF and VEGFR inhibitors on adipocyte-associated thermogenic activity, UCP1 levels and norepinephrine (NE)-induced thermogenic metabolism were measured. Animals were anaesthetized with pentobarbital to prevent shivering thermogenesis^36^. Under this condition, injection of NE would lead to activation of non-shivering thermogenesis from adipose tissues. VEGF and anti-VEGFR2 blockades as well as sunitinib significantly inhibited the beige transition and UCP1 expression in CL-treated gWAT (Fig. 3a,c,d). In particular, VEGFR2 blockade significantly inhibited UCP1 expression. Consistent with inhibition of UCP1 expression and the beige phenotype, the VEGFR2-specific antibody significantly inhibited the CL-induced non-shivering thermogenesis (Fig. 3e). Treatment of with these VEGF inhibitors did not significantly alter food intake, fat mass and lean compositions as measured by a magnetic resonance imaging (Supplementary Fig. 3d,e). To exclude the possibility that VEGF directly acted on adipocytes, we treated adipocytes with VEGF *in vitro*. VEGF did not augment UCP1 expression in white adipocytes, excluding the autonomous effect of VEGF on adipocytes (Supplementary Fig. 1h). These data provide evidence that the VEGF-VEGFR2 signalling system is crucially involved in modulating adipocyte metabolic functions.

### Endothelial knockout of VEGFR2 inhibits the WAT-beige transition

**Figure 4:**
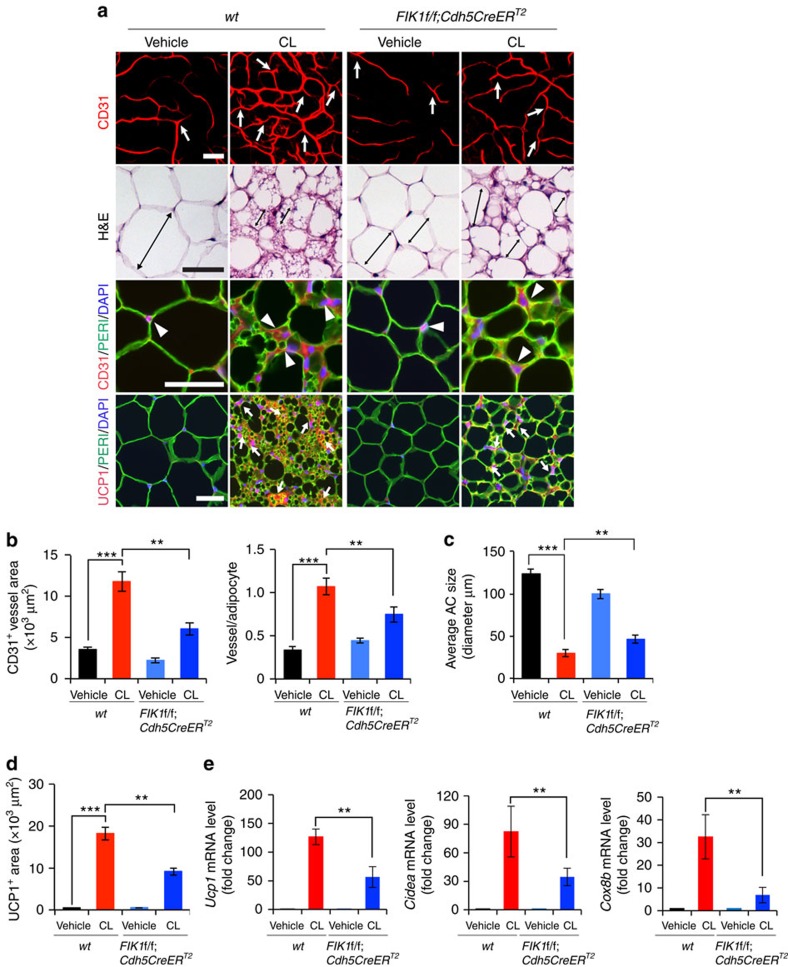
Impaired CL-induced angiogenesis and beige transition in EC *Vegfr2*-specific eliminated mice. (**a**) Microvessels (CD31^+^ red), adipocyte morphology (H&E; perilipin^+^ green), and UCP1 expression (UCP1^+^ red) of vehicle- and CL-treated gWAT in wt and *Flk1f/f;Cdh5CreER*^*T2*^ mice. White arrows and arrowheads in upper three panels point to microvessels. Arrows in the lowest row of panels indicate UCP1-positive signals. Double-arrowed bars mark adipocyte diameter. *n*=5 for each wt and *Flk1f/f;Cdh5CreER*^*T2*^ group. (**b**) Quantification of microvessel density in vehicle- and CL-treated wt and *Flk1f/f;Cdh5CreER*^*T2*^ mice (*n*=10 random fields; *n*=5 mice for each group). (**c**) Quantification of average adipocyte size (>30 adipocytes/field; *n*=10 random fields; *n*=5 mice for each group). (**d**) Quantification of UCP1-positive signals (*n*=10 random fields; *n*=5 mice for each group). (**e**) qPCR quantification of mRNA expression levels *Ucp1, Cidea, Cox8b, Pgc1α* and *Prdm16* in vehicle- and CL-treated total gWAT samples in wt and *Flk1f/f;Cdh5CreER*^*T2*^ mice. (*n*=10 samples for each group). All scale bars, 50 μm. **P<0.01; ***P<0.001 by two-sided unpaired *t*-test. Data presented as mean±s.e.m.

To further strengthen our conclusions of endothelial VEGFR2-modulated adipocyte functions, we specifically deleted VEGFR2 in ECs by crossing the *Flk1f/f* mouse strain with *Cdh5CreER*^*T2*^ and *Tie2CreER*^*T2*^ strains to generate *Flk1f/f;Tie2CreER*^*T2*^ and *Flk1f/f;Cdh5CreER*^*T2*^ strains, followed by induction with tamoxifen treatment for 5 days. To ensure that EC-specific deletion of the *Vegfr2* gene, we localized VEGFR2 expression in WAT. VEGFR2 expression in iWAT and gWAT was restricted to vascular ECs (Supplementary Fig. 6a). Food intake and body mass index (BMI) in CL- and vehicle-treated *Flk1f/f;Cdh5CreER*^*T2*^ mice were indistinguishable from those of wt mice (Supplementary Fig. 4a). CL-induced angiogenesis in gWAT and iWAT was significantly attenuated in *Flk1f/f;Cdh5CreER*^*T2*^ mice as compared with that in wt mice (Fig. 4a,b and Supplementary Fig. 4c,d). CL-induced browning and UCP1 levels of gWAT was significantly impaired in *Flk1f/f;Cdh5CreER*^*T2*^ mice as compared with controls (Fig. 4c-e). In addition, *Cidea* and *Cox8b*, browning markers, were also significantly decreased in *Flk1f/f;Cdh5CreER*^*T2*^ mice (Fig. 4e). CL-augmented CD31^+^ vessel density of iBAT in *Flk1f/f;Cdh5CreER*^*T2*^ mice was decreased (Supplementary Fig. 4f,g) without affecting CL-induced UCP1 expression (Supplementary Fig. 4h), indicating that the VEGF-VEGFR2 signalling is involved in browning of WAT.

To further validate these findings, we performed the same-setting experiments in *Flk1f/f;Tie2CreER*^*T2*^, although deletion of *Vegfr2* gene in this mouse strain might be less specific for endothelial cells. Food intake and BMI in CL-and vehicle-treated *Flk1f/f;Tie2CreER*^*T2*^ mice were indistinguishable from those of wt mice (Supplementary Fig. 6b). *Vegfr2* expression of ECs in WAT was significantly decreased in *Flk1f/f:Tie2CreER*^*T2*^ mice (Supplementary Fig. 6c). CL-induced gWAT angiogenesis was significantly impaired in *Flk1f/f;Tie2CreER*^*T2*^ mice (Supplementary Fig. 5a,b), supporting the notion that the VEGFR2 signalling is essential for CL-induced adipose angiogenesis. Similarly, CL-induced angiogenic phenotype was also impaired in iWAT and iBAT (Supplementary Fig. 6d,e,g,h). *Flk1f/f:Tie2CreER*^*T2*^ mice also markedly attenuated the CL-induced gWAT beige phenotype (Supplementary Fig. 5a,c). Consistent with phenotypic alterations, UCP1 expression in gWAT and iWAT was largely ablated in *Flk1f/f;Tie2CreER*^*T2*^ mice (Supplementary Fig. 5a,d,e and Supplementary Fig 6d,f, j). Conversely, UCP1 expression in iBAT remained unchanged in wt and *Flk1f/f;Tie2CreER*^*T2*^ mice (Supplementary Fig. 6g,i,j). The CL-induced browing/beige markers in gWAT, including UCP1, CIDEA, COX8B, PGC-1α and PRDM16, were also significantly abrogated in *Flk1f/f;Tie2CreER*^*T2*^ mice (Supplementary Fig. 5e). To exclude the possibility that deletion of *Vegfr2* would have a direct impact on β3-adrenoceptor expression levels, we quantitatively measured β3-adrenoceptor mRNA levels of vehicle- and CL-treated gWAT and found no difference between wt and *Flk1f/f;Tie2CreER*^*T2*^ mice (Supplementary Fig. 5f). The downstream signalling mediator cAMP remained at the similar levels of CL-treated gWAT obtained from wt and *Flk1f/f;Tie2CreER*^*T2*^ mice (Supplementary Fig. 5g).

It seemed that attenuation of browning of WAT in *Flk1f/f;Cdh5CreER*^*T2*^ mice was less robust as compared that in *Flk1f/f;Tie2CreER*^*T2*^ mice. However, the knockout efficiency in *Flk1f/f;Cdh5CreER*^*T2*^ mice was lower than that in *Flk1f/f;Tie2CreER*^*T2*^ mice (Supplementary Fig. 4b and Supplementary Fig. 6c). In general, the knockdown efficiency in both mouse strains were well correlated with their phenotypic changes. These findings show that VEGFR2-activated ECs through a paracrine mechanism modulate adipocyte functions.

### Gain-of-VEGF-function induces a beige phenotype

**Figure 5:**
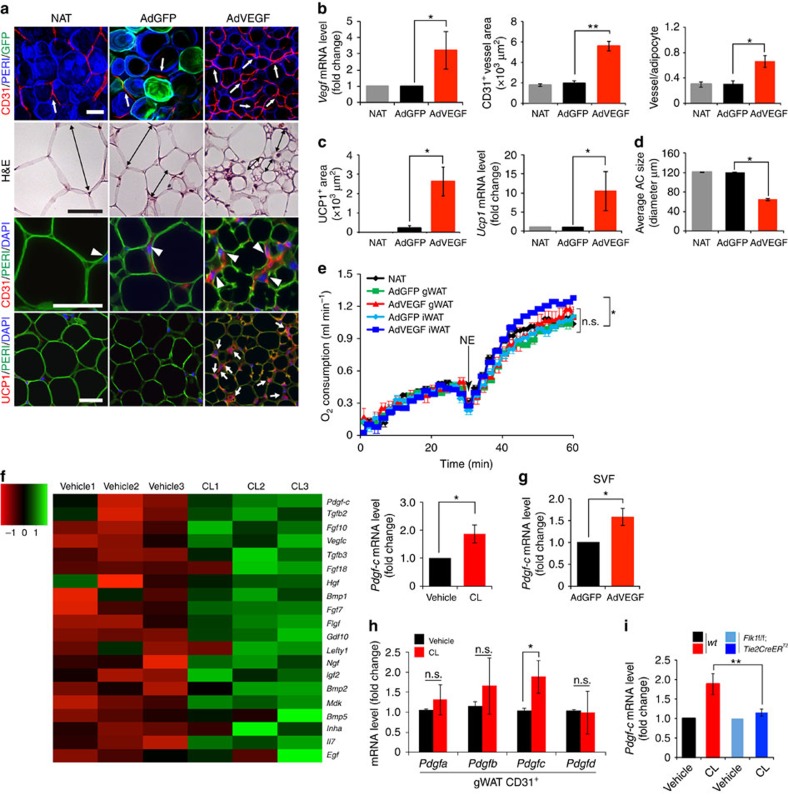
Microarray and effect of AdVEGF delivery on adipose angiogenesis and CL-induced PDGF-C expression during beige transition. (**a**) Histological images of microvessels (CD31^+^ red, white arrows and arrow heads), adipocyte morphology (H&E, double-arrowed bars, perilipin+green), and UCP1 (UCP1^+^ red, white arrows) in mice that were treated with AdGFP and AdVEGF for 10 days. The non-adenovirus-treated (NAT) group served as a control. *n*=5 mice for NAT group, *n*=10 mice for each AdGFP and AdVEGF group. (**b**) Quantification of *Vegf* mRNA expression levels in NAT- AdGFP- and AdVEGF-treated total gWAT. Quantification of microvessel density in NAT- AdGFP- and AdVEGF-treated (*n*=10 random fields; *n*=5 mice for NAT and *n*=10 for each AdGFP and AdVEGF group). (**c**) Quantification of UCP1-positive signals (*n*=10 random field; *n*=5 mice for NAT group and *n*=10 for each AdGFP and AdVEGF group) and quantification of *Ucp1* mRNA expression levels by qPCR (*n*=10 samples for each group). (**d**) Quantification of average adipocyte size (>30 adipocytes/field; *n*=10 random fields; *n*=5 mice for NAT group and *n*=10 for each AdGFP and AdVEGF group). (**e**) Norepinephrine-stimulated non-shivering thermogenesis in AdGFP or AdVEGF-treated gWAT of mice (*n*=5 mice for each group). NE, norepinephrine. (**f**) Hierarchical clusters of top 20 growth factors and cytokines from genome-wide microarray analysis (*n*=3 samples for each group). qPCR analysis of *Pdgf-c* mRNA expression levels of vehicle- and CL-treated gWAT SVF (*n*=5 samples for each group). (**g**) Quantification of *Pdgf-c* mRNA expression levels in SVF by qPCR in AdGFP- and AdVEGF-treated total gWAT (*n*=10 samples). (**h**) QPCR analysis of *Pdgf-a*, *-b*, *-c*, and *-d* expression levels in total vehicle- and CL-treated CD31^+^ EC fraction from gWAT. (**i**) *Pdgf-c* mRNA expression levels in vehicle- and CL-treated total gWAT of wt and *Flk1f/f;Tie2CreER*^*T2*^ mice. All scale bars, 50 μm. *P<0.05; **P<0.01 by two-sided unpaired *t*-test. Data presented as mean±s.e.m. n.s., not significant.

We next carried out gain-of-function experiments by delivering AdVEGF to WATs. As shown with GFP (green fluorescent protein)^+^ adenoviral particles, delivery of AdGFP to gWAT and iWAT led to and expression of GFP (Fig. 5a and Supplementary Fig. 7a). As expected, delivery of AdVEGF to gWAT resulted in a high expression level of VEGF that induced threefold increase of CD31^+^ microvessel density (Fig. 5a,b and Supplementary Fig. 7a,b). Transduction of gWAT with AdVEGF also led to a beige phenotype by increasing intracellular density of organelles, reducing adipocyte sizes and expression of UCP1 (Fig. 5a,c,d and Supplementary Fig. 7a,c). The similar findings have also been described by others^37^. Despite the browning of gWAT by AdVEGF transduction, thermogenesis-related metabolism remained the same as that in non-transduced control mice (Fig. 5e), indicating that browning of gWAT had no significant impact on overall energy expenditure. In contrast, delivery of AdVEGF to iWAT significantly increased global thermogenesis (Fig. 5e). These findings also validate the global impact of browning of subcutaneous WAT on thermogenesis as recently described by others^38^.

### VEGFR2 activation induces endothelial PDGF-CC expression

Knowing that VEGF-activated angiogenic vessels provide instrumental signals that regulate adipocyte functions via a paracrine mechanism, we focused our subsequent studies on identification of possible VEGF-induced EC-derived factors that activate the beige phenotype. For this reason, we performed mouse genome-wide Affymetrix gene expression analysis on the SVF of CL- and vehicle-treated gWAT. Hierarchical clustering of growth factors and cytokines showed that *Pdgf-c* was one of the upregulated growth factors in the CL-treated SVF of gWAT (Fig. 5f). Increased *Pdgf-c* expression levels were confirmed by quantitative PCR (qPCR) analysis (Fig. 5f). Similarly, *Pdgf-c* expression was significantly upregulated in SVF of AdVEGF-transduced gWAT (Fig. 5g). Moreover, *Pdgf-c* expression was limited to the CD31^+^ EC fraction of gWAT and CL-treatment markedly augmented *Pdgf-c* expression (Fig. 5h). Importantly, CL-induced *Pdgf-c* expression was significantly abrogated in *Flk1f/f;Tie2CreER*^*T2*^ mice (Fig. 5i). These findings demonstrate that CL-induced endothelial expression of PDGF-C is dependent on activation of the VEGFR2 signalling pathway.

### Genetic deletion of *Pdgf-c* gene impairs a beige phenotype

**Figure 6:**
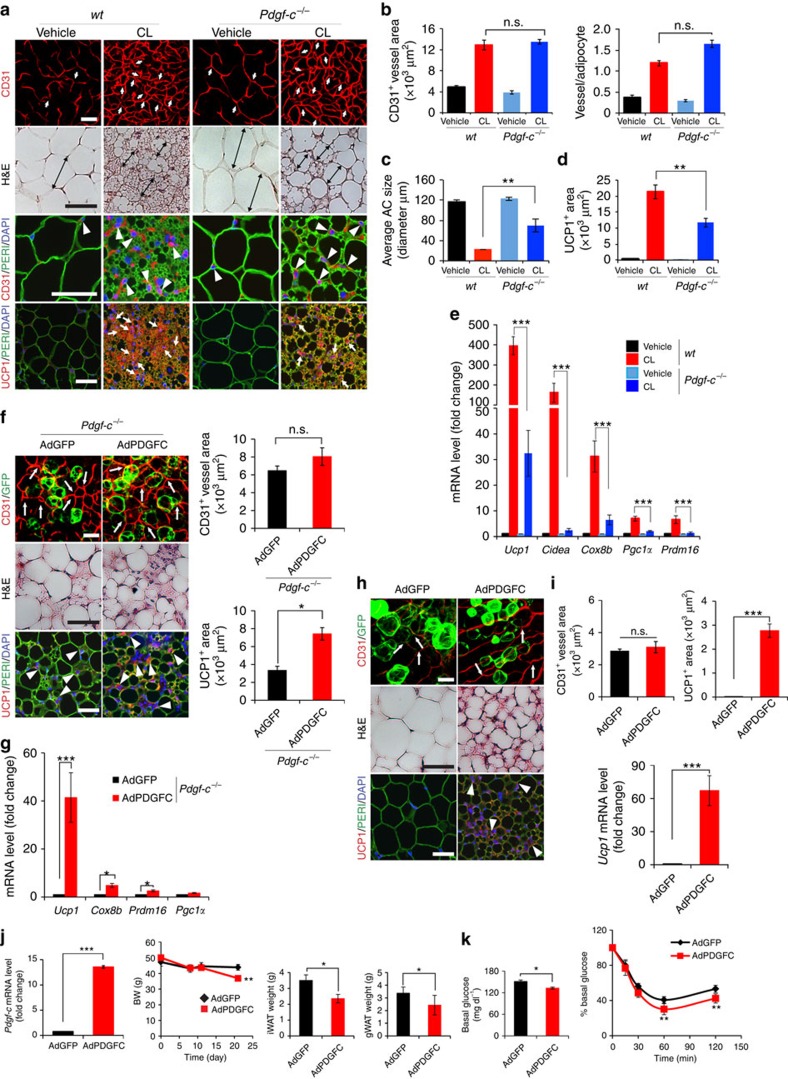
Impaired CL-induced beige transition in *Pdgf-c*^*−/−*^ mice and gain-of-function by delivery of AdPDGFC. (**a**) Histological images of microvessels (CD31^+^ red, white arrows and arrow heads), adipocyte morphology (H&E, double arrowed bars, perilipin+green), and UCP1 (UCP1^+^ red, white arrows). *n*=5 mice for each wt and *Pdgf-c*^*−/−*^group. (**b**) Quantification of microvessel density in vehicle- and CL-treated gWAT in wt and *Pdgf-c*^*−/−*^ mice. (*n*=10 random fields; *n*=5 mice for each group). (**c**) Quantification of average adipocyte size (>30 adipocytes/field; *n*=10 random fields; *n*=5 mice for each group). (**d**) Quantification of UCP1-positive signals (*n*=10 random fields; *n*=5 mice for each group). (**e**) qPCR quantification of browning markers' expression levels in vehicle- and CL-treated total gWAT samples in wt and *Pdgf-c*^*−/−*^ mice. (*n*=10 samples for each group). (**f**) Delivery of AdPDGFC to gWAT of CL-injected *Pdgf-c*^*−/−*^ mice. Arrows point to CD31^+^ microvessels and arrowheads point to UCP1-positive signals. Quantification of CD31^+^ and UCP1^+^ signals (*n*=10 randomized fields). *n*=8 mice for each wt and *Pdgf-c*^*−/−*^ group. (**g**) qPCR quantification of browning markers' expression levels in AdGFP- and AdPDGFC-injected gWAT of CL-treated *Pdgf-c*^*−/−*^mice (*n*=12 samples for each group). (**h**) Delivery of AdPDGFC or AdGFP to gWAT of NOD-SCID mice. Arrows point to CD31^+^ microvessels and arrowheads point to UCP1-positive signals. (**i**) Quantification of CD31^+^ and UCP1^+^ signals (*n*=10 randomized fields; *n*=8 mice for each group). (**j**) *Pdgf-c* expression level in iWAT by delivery of AdPDGFC, body weight curve and adipose depot weights of HFD-induced obese mice receiving AdGFP and AdPDGFC treatment (*n*=6 animals for each group). (**k**) Insulin tolerance of AdGFP- and AdPDGFC-treated HFD-induced obese mice (*n*=10 animals for each group). The Mann–Whitney test. All scale bars, 50 μm. *P<0.05; **P<0.01; ***P<0.001 by two-sided unpaired *t*-test. Data presented as mean±s.e.m. n.s., not significant.

To assess functional involvement of PDGF-CC in CL-induced beige transition, *Pdgf-c* gene was genetically eliminated in mice (*Pdgf-c*^*−/−*^ mice). Food intake and BMI in CL- and vehicle-treated *Pdgf-c*^*−/−*^ mice were indistinguishable from those of wt mice (Supplementary Fig. 8a,b). CL-induced angiogenesis in gWAT, iWAT and iBAT remained unaffected by knocking out *Pdgf-c* (Fig. 6a,b and Supplementary Fig. 8c,d), supporting the fact that PDGF-CC acts as a downstream mediator of the CL-induced VEGF-VEGFR2 signalling system. However, removal of *Pdgf-c* significantly impaired the CL-induced beige phenotype and UCP1 expression in gWAT (Fig. 6a,c,e) and iWAT (Supplementary Fig. 8c). Unlike beige tissues, UCP1 expression in iBAT remained similar levels in CL-treated wt and *Pgdf-c−/−* mice (Supplementary Fig. 8d,e). To validate these findings, we analysed a panel of beige- and BAT-related transcription factors and enzymes. CL-induced expression levels of CIDEA, COX8B, PGC-1α and PRDM16 were all significantly abrogated in *Pdgf-c*^*−/−*^ gWAT as compared with wt control gWAT (Fig. 6e). Consistent with phenotypic changes, CL-stimulated *Pdgf-c*^*−/−*^ mice showed impaired NE-stimulated non-shivering thermogenesis as compared with CL-stimulated wt mice (Supplementary Fig. 8f). Without CL treatment, wt and *Pdgf-c*^*−/−*^ exhibited nearly identical non-shivering theromogenesis in response to NE stimulation (Supplementary Fig. 8f). These data demonstrate that *Pdgf-c*^*−/−*^ mice exhibited impairment of non-shivering thermogenesis, most likely due to loss of the ability of browning WAT. In addition, glucose intolerance in CL-treated *Pdgf-c*^*−/−*^ mice was significantly increased as compared with that of CL-treated wt mice, whereas glucose levels remained similar in vehicle-treated *Pdgf-c*^*−/−*^ and wt mice (Supplementary Fig. 8g). Again, impairment of browning WAT is likely responsible for increased glucose intolerance in *Pdgf-c*^*−/−*^ mice.

Delivery of AdPDGFC to gWAT and iWAT in CL-treated *Pdgf-c*^*−/−*^ mice rescued UCP1 mRNA and protein expression, and induced a beige phenotype in these depots, without affecting angiogenesis (Fig. 6f,g). Similarly, AdPDGFC also induced a beige phenotype in gWAT (Fig. 6h,i) and iWAT (data not shown) of NOD mice. In principle, the findings in *Pdgf-c*^*−/−*^ mice were of the similar phenotype as seen with *Flk1f/f;Cdh5CreER*^*T2*^ and *Flk1f/f;Tie2CreER*^*T2*^ mice. Thus, these data demonstrate that EC-derived PDGF-CC partly mediates the CL-VEGF-VEGFR2-induced paracrine activation beige adipocytes.

### AdPDGFC improves glucose tolerance in HFD-obese mice

It is known that obese animals are catecholamine resistant and exhibit compromised expression of adrenergic receptors^39^,^40^. Thus, regulation of VEGF expression and subsequent expression levels could be impaired in high-fat diet (HFD) -induced obese mice. Indeed, VEGF and PDGF-CC expression levels in iWAT were markedly reduced in HFD-induced obese mice compared with control lean mice (Supplementary Fig. 6k). To further study the pathophysiological relevance, AdPDGFC was delivered to iWAT and gWAT in HFD-fed obese mice (BMI, 0.49±0.07). Noteworthy, AdPDGFC delivery resulted in significant weight reduction of iWAT and gWAT relative to AdGFP control-treated obese mice (Fig. 6j). Insulin tolerance test showed that AdPDGFC-treated obese mice exhibited significantly improved glucose clearance as compared with controls (Fig. 6k). Consistently, delivery of AdPDGF-C to mice also increased UCP1 mRNA and protein expression and energy expenditure (Supplementary Fig. 8h). These findings indicate that PDGF-CC in adipose tissues improves global insulin sensitivity in HFD-induced obese animals.

### PDGF-CC stimulates UCP1 expression in mouse and human PDGFR-α^+^ cells

**Figure 7:**
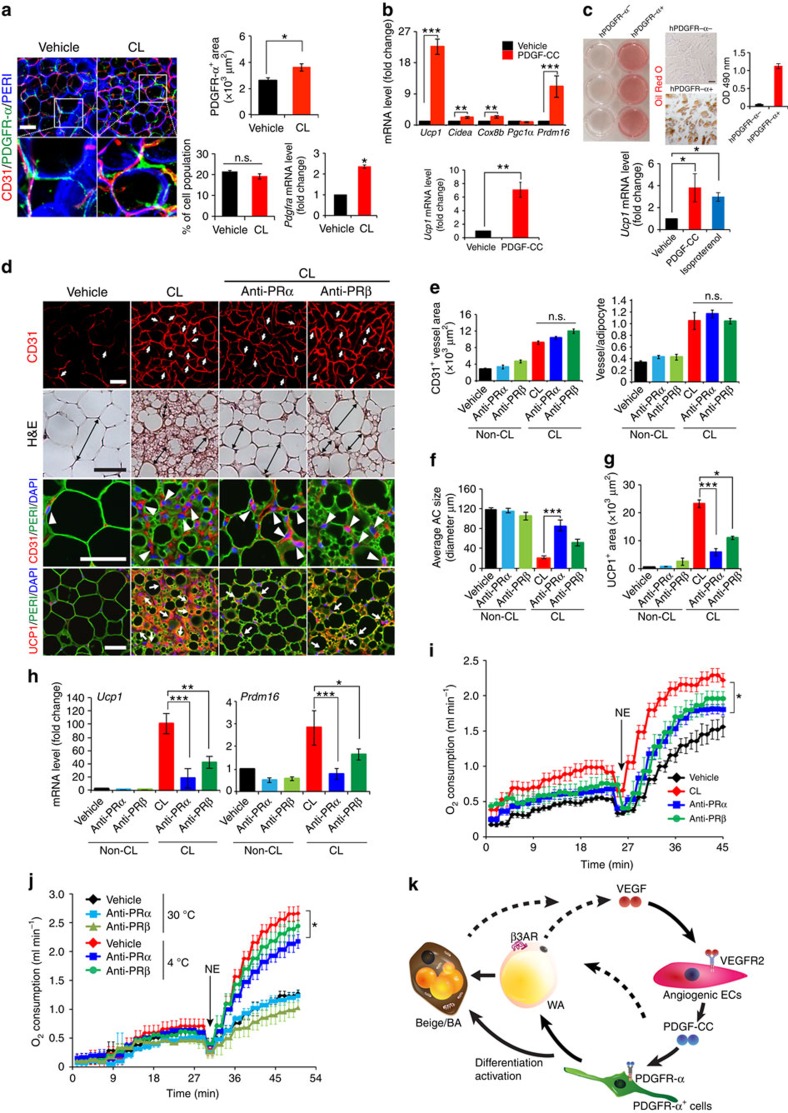
PDGF-CC promotes adipose progenitor cell differentiation toward a beige phenotype and PDGFR-α blockade CL-induced beige transition. (**a**) Localization of PDGFR-α^+^ cells, the quantification, FACS analysis, and *Pdgfra* mRNA expression in vehicle- and CL-treated gWAT of wt mice (*n*=4 for staining and *n*=6 for FACS; *n*=10 for quantification; *n*=6 for qPCR). (**b**) qPCR analysis of browning markers' expression levels by PDGF-CC stimulation in differentiated gWAT-PDGFR-α^+^ cell populations. (**c**) Oil Red O staining and qPCR analysis (*n*=9 samples for each group) in human differentiated PDGFR-α^−^ and PDGFR-α^+^ cells. (**d**) Histological images of microvessels (CD31^+^ red, white arrows and arrow heads), adipocyte morphology (H&E, double arrowed bars; perilipin+green), and UCP1 (UCP1^+^ red, white arrows). (**e**) Quantification of microvessel density in vehicle- and CL-treated gWAT in wt mice that received PRα and PRβ blockade treatment (*n*=10 random fields; *n*=8 mice for each group). (**f**) Quantification of average gWAT adipocyte size (>30 adipocytes/field; *n*=10 random fields; *n*=8 mice for each group). (**g**) Quantification of UCP1-positive signals (*n*=10 random fields; *n*=8 mice for each group). (**h**) qPCR quantification of browning markers' expression levels in gWAT from wt mice that received various treatments. (**i**) Norepinephrine-stimulated non-shivering thermogenesis in PRα and PRβ blockade- and vehicle-treated mice that received CL or buffer (*n*=5 mice for each group). NE, norepinephrine. (**j**) Norepinephrine-stimulated non-shivering thermogenesis in PRα and PRβ blockade- and vehicle-treated mice that had been exposed to 4 or 30 °C (*n*=5 mice for each group). (**k**) Schematic diagram of paracrine regulatory mechanisms by which the VEGF-VEGFR2 and PDGF-CC-PDGFR-α signalling systems cohesively and reciprocally control adipose endothelial cell-adipocyte crosstalk, leading to the transition of beige cell differentiation from PDGFR-α^+^ cells in WAT. AR, adrenoceptor; BA, brown adipocyte; WA, white adipocyte. All scale bars, 50 μm. *P<0.05; **P<0.01; ***P<0.001 by two-sided unpaired *t*-test. Data presented as mean±s.e.m. n.s., not significant.

Since PDGF-CC is known to bind to and activate PDGFR-αα homodimers and PDGFR-αβ heterodimers to induce biological activities^16^,^17^,^18^, we next studied the role of these PDGFRs in mediating the PDGF-CC-induced browning beige phenotype. First, we localized PDGFR-α expression in vehicle- and CL-treated gWAT and isolated the CD31^−^, PDGFR-α^+^, CD34^+^ and Sca1^+^ progenitor cells (PDGFR-α^+^ cells). PDGFR-α^+^ signals were not in vascular cells and the strong signals were not seen in mature adipocytes (Fig. 7a) as previously described^41^,^42^. Moreover, CL could further elevate the expression level of PDGFR-α (Fig. 7a). The increase of PDGFR-α was unlikely due to the increase PDGFR-α cell numbers by judging the FACS analysis, which did not show a difference between vehicle- and CL-treated gWAT (Fig. 7a).

Consistent with our findings, a study shows that adipose PDGFR-α^+^ cells can differentiate into white or brown-like adipocytes^41^. We isolated adipose PDGFR-α^+^ cells from gWAT and studied their ability of differentiation into mature adipocytes in response to PDGF-CC (Supplementary Fig. 9a). In response to PDGF-CC stimulation, PDGFR-α became significantly activated by phosphorylation in differentiated PDGFR-α^+^ cells after 5 min (Supplementary Fig. 9b). Upon differentiation into ‘mature' adipocytes, these cells seem to retain PDGFR-α expression at low levels and respond to PDGF-CC stimulation. Thus, upregulation of UCP1 expression by PDGF-CC may also occur in mature adipocytes. Delivery of AdPDGFC to gWAT also induced activation of PDGFR-α *in vivo* (Supplementary Fig. 9c). PDGF-CC stimulation resulted in an approximate 25-fold and 7-fold increase of the *Ucp1* expression in differentiated and undifferentiated cells, respectively (Fig. 7b), suggesting that PDGFR-α is involved in activation of a beige phenotype in these differentiated cells. Consistently, expression levels of several commonly used activated brown and beige adipocytes markers, including PRDM16, CIDEA and COX8b, were all significantly increased in response to PDGF-CC stimulation in the differentiated cells (Fig. 7b).

To translate our findings to clinical relevance, we isolated PDGFR-α^+^ and PDGFR-α^−^ cell populations from human healthy subcutaneous adipose tissues (Supplementary Fig. 9d). Importantly, human PDGFR-α^+^, but not PDGFR-α^−^, cells could differentiate into adipocytes that contained high contents of Oil Red O^+^ lipids (Fig. 7c). Further, stimulation of differentiated PDGFR-α^+^ adipocytes with PDGF-CC significantly increased *Ucp1* expression (Fig. 7c). These findings link our preclinical findings to human relevance showing that PDGFR-α^+^ cells in human adipose tissues are capable to differentiate into mature adipocytes that undergo a UCP1^+^ browning transition in the presence of PDGF-CC.

### PDGFR inhibitors attenuate a beige transition and thermogenesis

To further delineate the functional impact of PDGFR-triggered signals in beige transition, anti-mouse PDGFR-α and PDGFR-β-specific neutralizing antibodies^43^,^44^ were employed in our *in vivo* studies. CL-treated mice together with these PDGF-specific blockades did not alter BMI, food intake and CL-induced angiogenesis (Fig. 7d,e and Supplementary Fig. 9e, f), validating the finding obtained from *Pdgf-c*^*−/−*^ mice and indicating that PDGFR is a downstream component of the VEGF-VEGFR2 signalling. Inversely, PDGFR-α blockade markedly attenuated the CL-induced beige transition (Fig. 7d,f), showing the essential role of the PDGFR-α signalling in the CL-induced beige transition *in vivo*. Similar to PDGFR-α blockade, PDGFR-β blockade also significantly inhibited the beige adipose phenotype, albeit the inhibitory effect was weaker compared with that of PDGFR-α blockade (Fig. 7d,f). Given the fact that PDGF-CC binds to PDGFR-α/-β heterodimers, it was perhaps not surprising that blocking PDGFR-β also led to significant inhibition of the beige phenotype. However, PDGFR-α blockade produced a more potent inhibitory effect on the browning transition compared with that in *Pdgf-c*-deficient mice, suggesting other members in the PDGF family might contribute to the activation of PDGFR-α. In concordance with suppression of the beige phenotype, PDGFR-α and -β blockades markedly suppressed *Ucp1* and *Prdm16* expression (Fig. 7d,g,h and Supplementary Fig. 9g). Similarly, NE-induced thermogenic metabolism was also significantly inhibited by PDGFR-α and -β blockades (Fig. 7i). These findings validate our conclusion that the PDGF-CC-PDGFR-α and -β system mediates the CL-VEGF-VEGFR2-induced beige phenotype and adipocyte functions.

Varcularization measured by CD31^+^ structures, vascular permeability and blood perfusion assessed by extravasation and perfusion with 70 kD and 2000, kD lysine-fixable dextran respectively in iWAT, gWAT and iBAT did not alter compared with the vehicle (Supplementary Fig. 10a−10c) despite suppression of browning phenotypes, treatment of PDGFR-α and PDGFR-β blockades. These findings exclude the possibility that alteration of blood perfusion is responsible for PDGF-CC-mediated browning phenotypes.

To relate these findings to physiological relevance, we studied the effects of PDGFR blockades on cold-induced activation of subcutaneous WAT and BAT. Under cold exposure, the beige transition only occurred in subcutaneous WAT but not in gWAT. Treatment with PDGFR-α or PDGFR-β blockade significantly repressed cold-induced UCP1 expression in iWAT without affecting adipose angiogenesis (Supplementary Fig. 11a,d). However, the PDGFR-α blockade produced more overwhelming inhibitory effects on UCP1 expression than PDGFR-β blockade. Consistent with UCP1 levels, treatment with PDGFR-α blockade significantly inhibited thermogenesis-related energy expenditure (Fig. 7j and Supplementary Fig. 11d). PDGFR-β blockade treatment only produced a modest, but insignificant inhibition of norepinephrine-induced thermogenesis. In contrast to browning iWAT, PDGFR-β or PDGFR-α blockade treatment had no significant impacts on UCP1 expression in iBAT (Supplementary Fig. 11c,d). To quantitatively measure, the *Ucp1* mRNA expression levels under various treated conditions, we performed the full set analysis of our findings. Again, *Ucp1* mRNA expression levels were significantly decreased in iWAT, but not in iBAT (Supplementary Fig. 11d). These findings demonstrate that browning of subcutaneous WAT is responsible for overall thermogenesis-related energy expenditure and the PDGFR-α signalling is crucial for modulation of this process.

## Discussion

Activation of β3-adrenoceptor by an agonist (such as CL), as shown in this work, led to an angiogenic switch in both BAT and WAT. Why would activation of BAT and beiging of WAT lead to a robust angiogenic phenotype? Would adipose microvessels simply play an adoptive role in providing the high oxygen demand by these metabolically active tissues? Is it possible that angiogenic ECs produce paracrine signals to control adipocyte function? Currently, these vasculature-related questions have not been addressed. We first showed β3-adrenergic activation-augmented angiogenesis is dependent on the VEGF-VEGFR2 signalling. It is unlikely that VEGF-B would play any role in our model system because its expression level remained unchanged and VEGF-B does not bind to VEGFR2. Both VEGF and VEGFR2 inhibitors virtually blocked the CL-induced beige WAT browning. EC-specific deletion of *Vegfr2* gene reproduced the same WAT browning defective phenotype, substantiating the VEGF-EC signalling system that controls WAT browning. VEGF-induced angiogenesis might contribute to the WAT browning via several mechanisms, including, but not limited: (1) increasing blood perfusion; (2) paracrine stimulation of adipocytes via secretion of soluble factors; (3) inducing progenitor cell differentiation; and (4) direct differentiation of ECs into beige-like cells. Our present data support (2) and (3) possibilities of the existence of a paracrine regulatory mechanism. VEGF-stimulated angiogenic ECs could alter gene expression profiles by producing an array of growth factors, cytokines, receptor signalling molecules and intracellular signalling components. These angiogenic EC-derived factors can act as paracrine signalling molecules on other cell types in the surrounding microenvironment.

In our genome-wide microarray analysis, we discovered that PDGF-CC, a relative new member in the PDGF family, was one of the angiogenic factors upregulated in the vascular fraction of beige adipose tissue. Emerging evidence shows that PDGF-CC is a multifarious growth factor that promotes angiogenesis, vascular remodelling, and neuronal survival^18^,^45^,^46^,^47^,^48^,^49^,^50^,^51^, but its role in modulating adipose tissue function is unknown. How could PDGF-CC modulate adipocyte functions? Would this factor directly act on mature WAT adipocytes and reprogram their beige-related functions? Would it be possible that PDGF-CC promotes proliferation and differentiation of adipose progenitor cells toward a beige-like phenotype? If so, which receptor is mediating this effect? In *Pdgf-c*^*−/−*^ mice, we first demonstrate that the CL-induced gWAT browning is largely abrogated by marked reduction of UCP1 and other beige-specific markers, leaving gWAT vascular density unaffected. These data validate the fact that PDGF-CC is the downstream factor of the VEGF-VEGFR2 system. Both PDGFR-α and -β blockades significantly inhibit UCP1 expression and the beige transition without affecting CL-induced angiogenesis, suggesting that PDGFR-α and -β are both involved. PDGF-CC binds and activates PDGFR-αα and -αβ dimeric receptor isoforms. We should emphasize that other PDGF ligands such as PDGF-BB may also contribute to CL-induced beige transition by activation of PDGFR-α and -β signalling. Surprisingly, PDGF-BB does not potently induce WAT browning *in vivo*. Although the mechanistic insight underlying differential effects of PDGF-CC and PDGF-BB is lacking, it is highly plausible that PDGFR-α and PDGFR-β transduce different signals that drive progenitor cell differentiation towards different lineages. This interesting possibility warrants future investigation.

We show that PDGF-CC promotes PDGFR-α^+^ progenitor cells differentiation towards ‘browning' adipocytes that express UCP1. In support of our findings, a recent study shows that isolated adipose PDGFR-α^+^ cells can differentiate into WAT or BAT adipocytes^41^. Upon differentiation into ‘mature' adipocytes, these cells seem to retain PDGFR-α expression at low levels and respond to PDGF-CC stimulation. Thus, UCP1 upregulation by PDGF-CC may also occur in mature adipocytes. Noteworthy, PDGF-CC-induced browning phenotype is independent from changes of blood perfusion, since PDGFR blockades treatments did not alter blood perfusion in various adipose depots. Thus, PDGF-CC may act as a driving factor that promotes PDGFR-α^+^ cells towards BAT differentiation. Our notion of inhibition of PDGFRβ signalling impairs WAT browning is surprising because of lacking PDGFRβ expression in preadipocytes^41^. One explanation, although not well approved, is that the PDGFR-β-triggered signalling is crucial for supplying of preadipocytes from pericytes. Indeed, PDGFR-β^+^ pericytes is an important reservoir for preadipocytes^52^. This interesting possibility warrants further investigation.

Gain-of-function experiments show that PDGF-CC-induced WAT browning have a profound impact on global changes of physiological functions. The fact that PDGF-CC-induced WAT browning increases glucose tolerance and insulin sensitivity in HFD-induced obese animals suggests that PDGF-CC-induced WAT browning may be considered for treatment of type 2 diabetes in human patients. Indeed, such a PDGF-CC-induced differentiation mechanism of brown-like adipocytes exists in humans as demonstrated in our present findings. Taken together, our data provide evidence that angiogenic vessels in the adipose tissues control adipocyte functions through a PDGF-CC-mediated paracrine mechanism (Fig. 7k). These findings may have therapeutic implications for the treatment of obesity and related metabolic diseases such as type 2 diabetes.

## Experimental procedures

### Animals

C57Bl/6 mice were obtained from the breeding unit of the Department of Microbiology, Tumor and Cell Biology, Karolinska Institute, Stockholm, Sweden. The genetic knockout mice used in our study include: β3-adrenoceptor-deficient (*Adrb3*^*−/−*^) mice in FVB/N background were kindly provided by Dr Bradford Lowell at the Beth Israel Deaconess Medical Center, Harvard Medical School; uncoupling protein 1-deficient (*Ucp1*^*−/−*^) mice in 129 background were kindly provided by Dr Barbara Cannon at the Stockholm University; platelet-derived growth factor-C-deficient (*Pdgf-c*^*−/−*^) mice in 129 background were generated as previously described^50^,^53^; the EC-specific VEGFR2-deficient mice in C57Bl/6 background was generated by crossing *Flk1flox/flox* mice (kindly provided by Dr Thomas N. Sato at the Graduate School of Biological Science, Nara Institute of Sciences and Technology, National University Corporation) with *Tie2CreER*^*T2*^ or *Cdh5CreER*^*T2*^ mice in C57Bl/6 background (generated by Dr Arnold Bernd and kindly provided by the European Mouse Mutant Archive and by Dr Ralf Adams at the Max Planck Institute for Molecular Biomedicine, Muenster, Germany. EC deletion of V*egfr2* was achieved by treating mice with tamoxifen (see below). All mouse studies were carried without randomization or blinding, and were approved by the Northern Stockholm Experimental Animal Ethical Committee.

### Cold exposure and treatments

Female C57Bl/6 mice (Charles River, Wilmington) at age between 7 and 8 weeks old were adapted at 18 °C for 1 week before exposure to 4 °C for 10 days, or to 30 °C for 10 days (*n*=8 mice/group per experiment). For β3-adrenoceptor agonist treatment, wt C57Bl/6 mice, *Adrb3*^*−/−*^ mice, *Ucp1*^*−/−*^ mice, and these wts were intraperitoneally (i.p.) injected with CL (1 mg kg^−1^ in 0.1 ml) once daily for 10 consecutive days. Mice were killed 24 h after the last treatment and iBAT and different WAT depots were dissected for further experimentation. For RNA and protein extraction, fresh tissues were immediately freeze in dry ice and stored in −80 °C until further use. A portion of fresh tissues was immediately dissected, fixed with 4% paraformaldehyde overnight, and subsequently used for histological and immunohistochemical analyses. Buffer-treated control (vehicle) animals served as controls (*n*=8 mice/group per experiment).

For pharmacological treatment, mice received treatment of CL or vehicle for 10 days (*n*=8 mice/group/experiment) and simultaneously received other therapeutic agents for 10 days that include: sunitinib (60 mg kg^−1^ gavage/once daily, total 10 times during the entire experimentation); anti-VEGF (2.5 mg kg^−1^, i.p., twice/week, 3–4 times during the entire experimentation); anti-VEGFR2 (20 mg kg^−1^, i.p., twice/week, 3–4 times during the entire experimentation, ImClone Systems); anti-PDGFR-α (10 mg kg^−1^, i.p., twice/week, 3–4 times during the entire experimentation, ImClone Systems); or anti-PDGFR-β (10 mg kg^−1^, i.p., twice/week, 3–4 times during the entire experimentation, ImClone Systems). These treatment regimens are known to effectively block their targeted signalling pathways^35^,^54^. To investigate if PDGFR blockades inhibited cold-induced browning phenotype, cold-exposed mice (*n*=8 mice/group/experiment) were i.p. treated with anti-PDGFR-α and anti-PDGFR-β antibodies during cold exposure for 10 days and 4 weeks (10 days for the browning gene expression analysis and 4 weeks for measurement of the metabolic rate). Metabolic rates were measured on the last day of treatment. Mice were analysed for the metabolism, followed by killing and tissue collection. Vehicle-treated mice using the same regimens served as a control group.

### Generation of *Pdgf-c^−/−^* and endothelial *Vegfr2^−/−^* mouse strains

*Pdgf-c*^−/−^ mice on a 129 background as previously described^50^,^53^ were backcrossed with mice on a C57Bl/6 background. For generation of the EC-specific *Vegfr2*^−/−^ mouse strain, floxed *Flk1* (*Vegfr2*) mice were crossed with either the *Cdh5CreER*^*T2*^ or the *Tie2CreER*^*T2*^ strain, and these transgenic strains were identified by genotyping using either ear or tail DNA and a PCR-based method. The specific primer sequences for genotyping are shown in Supplementary Table 1. Deletion of *Vegfr2* in the *Flk1f/f;Cdh5CreER*^*T2*^ and *Flk1f/f;Tie2CreER*^*T2*^ mice was achieved by tamoxifen induction (2 mg per mouse, i.p. once daily for 5 consecutive days). At the end of day 3 after the last tamoxifen injection, mice received CL treatment for 10 consecutive days.

### Materials

CL316,243 (CL) was purchased from Tocris Bioscience (Bristol, UK). Bovine serum albumin, collagenase II, dexamethasone (DEX), insulin, 3-isobutyl-1-methylxanthine (IBMX), isoproterenol and tamoxifen were purchased from Sigma-Aldrich (St Louis, MO, USA). Recombinant mouse and human PDGF-CC was obtained from R&D Systems Inc. (Minneapolis, MN, USA). Primer sequences for all PCR and qPCR experiments are listed in Supplementary Table 1. Hepes, DMEM and lysine-fixable fluorescein dextran at molecular weight of 70 kD (D1822) or 2,000 kD (D7137) were purchased from Thermo Fisher Scientific Inc (Waltham, MA, USA). Antibody sources are: a rat anti-mouse Endomucin was purchased from eBioscience (Cat No. 14-5858-85; 1:400); a rabbit anti-mouse UCP1 (ab 10983; 1:200) and a rabbit anti-mouse Prohibitin (ab28172; 1:100) were purchased from Abcam (Cambridge, UK); a rat anti-mouse CD31 was purchased from BD Pharmingen (553370, Franklin Lakes, NJ, USA), or from Angio-Protemomic (mAP-0032, Boston, MA, USA); a PE-streptavidin was purchased from BD Pharmingen (554061); a mouse anti-human CD31 was purchased from Dako Agilent Technologies Company (GA610, Santa Clara, CA, USA); an anti-VEGFR1-Alexa Fluor 488 (FAB4711G); and a human PDGFR-α biotinylated antibody (BAF322) were purchased from the R&D Systems Inc. An anti-VEGFR2-Alexa Fluor 647 was purchased from BioLegend (121916, San Diego, CA, USA); an anti-mouse β-actin was purchased from Cell Signaling Technology (3700S, Danvers, MA, USA); an anti-mouse CD34-FITC antibody was purchased from eBioscience (Cat No. 11-0341-81, San Diego, CA, USA); an anti-mouse Ly6A/E (Sca1)-PE antibody was purchased from eBioscience (Cat No. 12-5981-81); and an anti-mouse p-PDGFR-α was purchased from Cell Signaling Technology (Cat No. 3164S); and an anti-mouse PDGFR-α (CD140a)-APC was purchased from eBioscience (Cat No. 14-1401-81). The rabbit anti-mouse VEGFR2 antibody was kindly provided by Dr Rolf Brekken at the Southwestern Medical Center, Dallas.

### Cell culture and proliferation assay

hTERT^+^-BCE endothelial cells were maintained in DMEM supplemented with 10% FBS, 100 units per ml penicillin and 100 μg ml^−1^ streptomycin^55^. Cells were treated with or without CL (0.33–330 μM) for 72 h. Proliferation was assayed using a MTS assay system (Promega, Madison, WI, USA). 3T3-L1 and 3T3F442A preadipocytes were cultured and maintained in DMEM supplemented with 10% FBS, 100 units per ml penicillin and 100 μg ml^−1^ streptomycin. Cell differentiation was induced with 850 nM insulin, 0.5 mM IBMX and 0.1 μM DEX for 48 h, followed by treatment with 850 nM insulin for 7–10 days. After 10 day-induction of differentiation, cells were treated with isoproterenol (10 μM), VEGF (100 ng ml^−1^), or vehicle for 4 h. UCP1 expression was determined by qPCR.

### Histology and immunohistochemistry

Paraffin-embedded tissue sections in 5 μm thickness were stained with haematoxylin and eosin (H&E) using our standard protocol^43^. For UCP1 and CD31 staining, the paraffin-embedded tissue sections were incubated using a rabbit anti-mouse UCP1 antibody (1:200; Abcam, Cambridge, UK) and a rat anti-mouse CD31 antibody (1:200), followed by staining with a mixture of secondary antibodies comprising an Alexa Fluor 555-labelled goat anti-rat antibody (1:200; Invitrogen, Carlsbad, CA, USA) and an Alexa Fluor 649-labelled goat anti-rabbit antibody (1:400; Jackson ImmunoResearch Laboratories Inc., West Grove, PA, USA). Positive signals were captured using a fluorescence microscope equipped with a Nikon DS-QilMC camera (Nikon Corporation, Tokyo, Japan). A rabbit anti-mouse VEGFR2 antibody (1:100 dilution in blocking buffer) was used for incubation at 4 °C overnight. Secondary fluorescent-conjugated antibodies, a goat anti-rabbit Alexa 555, Invitrogen, A21434; 1:400 dilution in blocking buffer was incubated at RT for 1 h.

### Whole-mount staining

Whole-mount staining was performed according to our previously published standard method^20^,^56^,^57^. Paraformaldehyde-fixed iWAT, gWAT and iBAT tissue samples were digested with proteinase K (20 μg ml^−1^) for 5 min and blocked with skim milk, followed by staining overnight at 4 °C with a rat anti-mouse CD31 antibody (1:200). Following rigorous rinsing in PBS, blood vessels were detected with an Alexa Fluor 555-labelled secondary antibody, mounted in Vectashield mounting medium (Vector Laboratories, Inc., Burlingame, CA, USA), and stored at −20 °C in darkness before examination under a Nikon C1 confocal microscope (Nikon Corporation). Captured images were further analysed using an Adobe Photoshop CS software program.

### RNA isolation, PCR and qPCR analyses

Total RNA was extracted from cells and tissues using TRIzol (Invitrogen) and GeneJET RNA Purification Kits according to the manufacturer's instructions. The total RNA was reversely transcribed and cDNAs were used for PCR and qPCR analyses using the primers listed in Supplementary Table 1.

### Protein extraction

Tissues were lyzed using the CellLytic MT Mammalian Tissue Lysis Extraction Regent (Sigma-Aldrich) with protease and phosphatase inhibitors. VEGF levels were determined using a sensitive ELISA kit according to the manufacturer's instruction (R&D Systems Inc.). For immunoblotting, the protein lysates were incubated with a specific primary antibody followed by an anti-mouse secondary antibody conjugated with IRDye 680RD (LI-COR, Lincoln, NE, USA; 1:15,000) or an anti-rabbit secondary antibody conjugated with IRDye 800CW (LI-COR, Lincoln, NE, USA; 1:15,000). Target proteins were detected using an Odyssey CLx system (LI-COR). Full-gel images are shown in Supplementary Fig. 12.

### ELISA

VEGF and cAMP levels were determined using a sensitive ELISA kit according to the manufacturer's instruction (MMV00 and KGE002B, R&D Systems Inc.).

### Microarrays

Genome-wide microarray analysis was used for our study. Microarray hybridization, scanning and analysis were performed Shanghai Biotechnology Corporation (Shanghai, China) using Affymetrix Mouse Gene 2.0 ST Array according to established their methods.

### Fractionation of MAF and SVF from WAT

Primary MAF and SVF from gWAT of C57Bl6 female mice at age between 4 and 5 weeks old were fractioned by a collagenase digestion method^58^. Tissues were removed, minced and were digested in a collagenase digestion solution (0.15% collagenase II, 10 mM HEPES and 5% bovine serum albumin in DMEM) for 0.5 h at 37 °C. During digestion, the suspension was triturated with vortex every 5 min and eventually quenched in ice for 5 min, followed by centrifugation at 300*g* for 5 min. The floating adipocytes were collected, washed with PBS and centrifuged at 300*g* for 10 min. The floating adipocytes were harvested as MAF. The remaining suspension was passed through a 100 μm mesh to remove undigested debris and effluents were centrifuged at 300*g* for 10 min. The SVF pellet was washed once with PBS. Cells in SVF were subjected to FACS for isolation of CD31^+^, PDGFR-α^+^, CD34^+^ and Scal1^+^ cell populations (PDGFR-α^+^). Isolated primary cell populations were cultured in DMEM medium supplemented with 20% FBS, 100 units per ml penicillin, 100 μg ml^−1^ streptomycin and 5 ng ml^−1^ FGF2 (R&D Systems Inc.). The PDGFR-α^+^ cells were differentiated by treatment with F12/DMEM containing 0.5 mM IBMX, 0.1 μM dexamethasone, 1 μM rosiglitazone and 1 μg ml^−1^ insulin for 4 days, following by treatment with 1 μg ml^−1^ insulin for 7–10 days. At day 10, the fully differentiated cells were stained with oil red O to visualize the lipid droplets and treated with isoproterenol (10 μM), mouse recombinant PDGF-CC (100 ng ml^−1^, R&D Systems Inc.), or vehicle for 4 h to assess UCP1 expression.

To investigate whether PDGF-CC plays an role in induction of UCP1 expression in undifferentiated PDGFR-α^+^ cells, the cells were treated with or without mouse recombinant PDGF-CC (100 ng ml^−1^, R&D Systems Inc.) in DMEM containing 10% FBS, 100 units/ml penicillin, and 100 μg ml^−1^ streptomycin for 7 days. The gene expression was measured by qPCR 7 days after the PDGF-CC treatment.

### Body composition and indirect calorimetry

Mouse body composition was measured using a magnetic resonance imaging technique (EchoMRI-700/100 Body Composition Anlyzer, Echo Medical Systems, Houston, TX). Norepinephrine (NE)-induced thermogenesis was determined by an assessment of oxygen consumption using an open-circuit indirect calorimeter INCA system (Somedic INCA, Hörby, Sweden) as the details previously described^21^,^36^. The Zirconium oxide sensors were calibrated with reference gases (18 and 25% O_2_ in N_2_) before the measurement. The mice were anaesthetized and the basal metabolic rate was measured before NE injection. All metabolic experiments were performed twice.

### Adenovirus delivery

For the gain-of-function and the rescue studies using various adenovirus vectors, wt, immune deficiency NOD-SCID IL2Rgammanull (NOD) mice, *Flk1f/f;Tie2CreER*^*T2*^ , and *Pdgf-c*^*−/−*^ mice were anaesthetized with Hypnorm (fentanyl citrate and fluanisone, VetaPharma, Leeds, UK) and midazolam (Dormicum, F. Hoffmann-La Roche Ltd, Basel, Switzerland) and gWAT or iWAT were injected with adenovirus expressing VEGF, PDGF-C, and PDGF-B (AdVEGF; AdPDGFC; AdPDGFB, 2 × 10^10^ viral particles per μl, 25 μl per injected site, three sites/adipose tissues, Vector Biolabs, Philadelphia, PA, USA) using 30G insulin needle. An adenovirus carrying green fluorescent protein (AdGFP, Vector Biolabs) was used as a negative control. For study the VEGF gain-of-function in adipose tissue, 25 μl per injected site (three sites/adipose tissues) of AdVEGF and AdGFP were injected into iWAT and gWAT in NOD mice. The adipose tissues were dissected after killing of mice on days 5, 10 or 21 after these infections. For metabolic measurement, AdGFP and AdVEGF (25 μl per injected site, three sites/adipose tissues) were injected into either iWAT or gWAT in C57Bl/6 mice for 14 days. After 14 days, the NE-induced metabolic rate of the mice was measured by the indirect calorimeter INCA system as described above. To investigate whether PDGF plays a key role in the browning of WATs, AdPDGFB, AdPDGFC and AdGFP as control were injected into iWAT and gWAT in NOD mice for 21 days. To investigate whether the gain of PDGF-C rescues CL-induced the phenotype of *Pdgf-c*^*−/−*^ mice, AdPDGFC and AdGFP were injected into iWAT and gWAT of CL-treated *Pdgf-c*^*−/−*^ mice and the treatment persisted for 15 days. For treatment of obese mice, AdPDGFC and AdGFP (25 μl per injected site, four sites per adipose tissues) were injected into iWAT and gWAT of 12 weeks HFD-fed- wt mice for 5, 10 and 21 days (5 days for the *Pdgf-c* gene expression analysis, 10 days for an insulin tolerance test and 21 days for observation of body weight), followed by intraperitoneal injection of insulin. Blood samples were harvested at different time points after insulin injection and subjected to a tolerance test according to our previously published method^59^.

### Oral glucose tolerance tests

Vechicle- and CL- treated wt and *Pdgf-c*^*−/−*^ mice were fasted for 6 h during the light phase with free access to water on a day after the last CL treatment. The blood from the tail vein was used to measure glucose levels using a glucometer (Accu-Chek, Aviva, Roche Diagnostics GmbH, Mannheim, Germany) immediately before and at 15, 30, 60 and 120 min after oral feeding with 1.5 mg glucose with 10 μl g^−1^-body weight.

### FACS analysis

SVF isolated from gWAT was incubated with an anti-CD31 (PECAM1)-PE antibody (1:100), an anti-VEGFR1-Alexa Fluor 488 antibody (1:50), an anti-VEGFR2-Alexa Fluor 647 antibody (1:50), an anti-CD34-FITC antibody (1:200), an anti-Ly6A/E (Sca1)-PE antibody (1:200) or an PDGFR-α (CD140a)-APC (1:100) antibody for 45 min on ice followed by flow cytometry using FACScan and CellQuest software (BD Biosciences, Franklin Lakes, NJ, USA). For analysis of VEGFRs, forward and side scatter gates were set and approximately, 10,000 events were analysed per sample using a CellQuest software.

### Isolation of the PDGF-α^+^ progenitors from human adipose tissues

Small pieces of subcutaneous WAT were collected from 25- to 53-year-old female subjects. SVF of subcutaneous WAT was fractionated using a collagenase-digestion-based method as in mouse WAT tissues. Cells from SVF were incubated with an anti-human CD31 antibody and a biotinylated anti-human PDGFR-α antibody for 45 min on ice, followed by incubation a PE-streptavidin and a donkey anti-mouse Alexa 488 secondary antibody, respectively. PDGFR-α^+^ and CD31^−^ human progenitor cells (hPDGFR-α^+^) were isolated by FACS sorting. The isolated PDGFR-α^+^ cells were cultured in F12/DMEM medium containing 5 ng ml^−1^ FGF2 and 20% FBS. The PDGFR-α^+^ cells were induced for differentiation with the F12/DMEM containing IBMX, dexamethasone, rosiglitazone and insulin for 4 days, following by treatment with insulin for 7 days. At day 10, the fully differentiated cells were stained with oil red O and treated with isoproterenol (10 μM), human recombinant PDGF-CC (100 ng ml^−1^, R&D Systems Inc.), or vehicle for 4 h. UCP1 expression was determined by qPCR. All human studies were approved by the Regional Ethics Committee of Stockholm. All participants provided informed written consent.

### Vascular permeability and perfusion

Lysine-fixable fluorescein dextran at molecular weight of 70 or 2,000 kD was injected into the tail vein of each mouse that received PDGFR-α, PDGFR-β or vehicle treatment as described in the main text. At 5 min after injection with 2,000 kD and 15 min after injection with 70 kD, mice were killed and various adipose depots were immediately fixed with 4% paraformaldehyde overnight and proceeded for further analyses by whole-mount staining.

### Statistical analysis

All data presented as mean±s.e.m. *P* values were deemed by two-sided Student's *t*-test or the Mann–Whitney test. Comparison of multiple groups was achieved using an ANOVA-SPSS software. *P<0.05 is considered significant.

### Data availability

Microarray data have been deposited in Gene Expression Omnibus (GEO) under accession code GSE55934. The authors declare that the data supporting the findings of this study are available within the article and its Supplementary Information files.

## Additional Information

**How to cite this article:** Seki, T. *et al*. Endothelial PDGF-CC regulates angiogenesis-dependent thermogenesis in beige fat. *Nat. Commun.* 7:12152 doi: 10.1038/ncomms12152 (2016).

## Supplementary Material

Supplementary Information Supplementary Figures 1-12 and Supplementary Table 1
