# Supplementary material for: Endothelial PDGF-CC regulates angiogenesis-dependent thermogenesis in beige fat
Source: Nat Commun. 2016 Aug 5;7:12152. doi: 10.1038/ncomms12152 (PMC4980448; doi:10.1038/ncomms12152)
Supplement: Supplementary Information — Supplementary Figures 1-12 and Supplementary Table 1 [file ncomms12152-s1.pdf]

SUPPLEMENTARY FIGURE

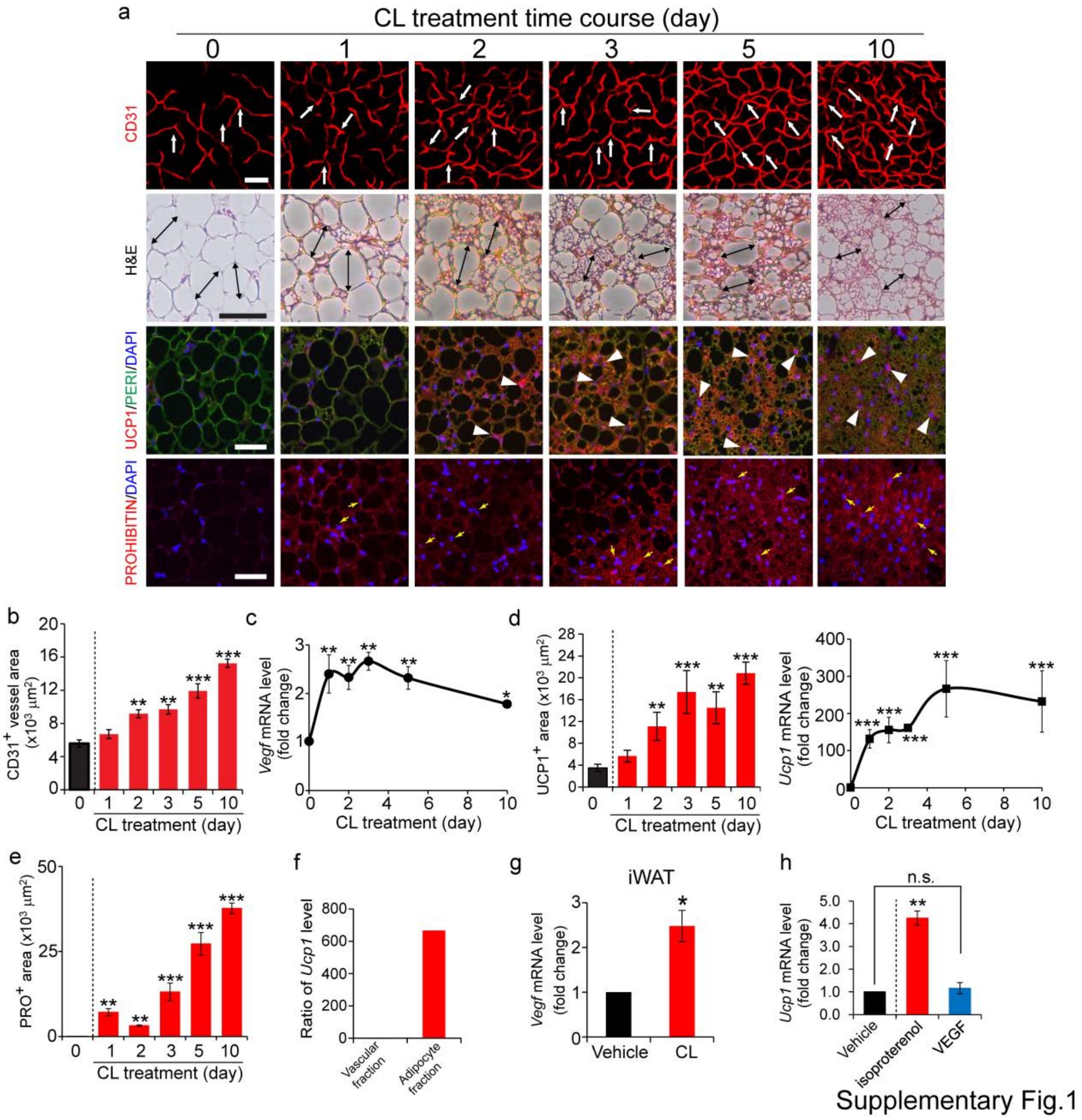

Supplementary Figure 1. Time course response to β3-adrenoceptor-dependent activation of adipose angiogenesis and the beige phenotype in visceral adipose tissue.

(a) Microvessels (CD31^{+} red), adipocyte morphology (H&E; Perilipin^{+} green), UCP1 expression and mitochondrial staining (Prohibitin^{+} red) of CL-treated

gWAT in mice. The buffer-treated control (vehicle) group served as a control. White arrows point to microvessels. Double arrowed bars mark adipocyte diameter. White arrowheads indicate UCP1 positive signals. Yellow arrows point to prohibitin positive signals. n = 5 mice for each group.

(b) Quantification of microvessel density in vehicle and CL-treated gWAT in *wt* mice (n = 10 random fields; n = 5 mice for each group).

(c) *Vegf* mRNA expression in CL-treated and vehicle -treated gWAT samples (n = 10 samples for each group).

(d) Quantification of UCP1 positive staining signals (n = 10 random fields; n = 5 mice for each group) and mRNA (n = 10 samples for each group).

(e) Quantification of prohibitin positive signals in vehicle - and CL- treated groups (n = 10 random fields; n = 5 mice for each group).

(f) qPCR quantification of *Ucp1* mRNA expression levels in vascular- and adipose-fraction.

(g) qPCR quantification of *Vegf* mRNA expression levels in vehicle - and CL-treated iWAT samples (n = 5 samples for each group).

(h) White adipocytes treated with isoproterenol, VEGF or vehicle. UCP1 expression was measured by qPCR. n.s. = not significant;

All scale bars, 50 μm. *$p < 0.05$; **$p < 0.01$; ***$p < 0.001$ by two-sided unpaired *t*-test. Data presented as mean ± s.e.m.

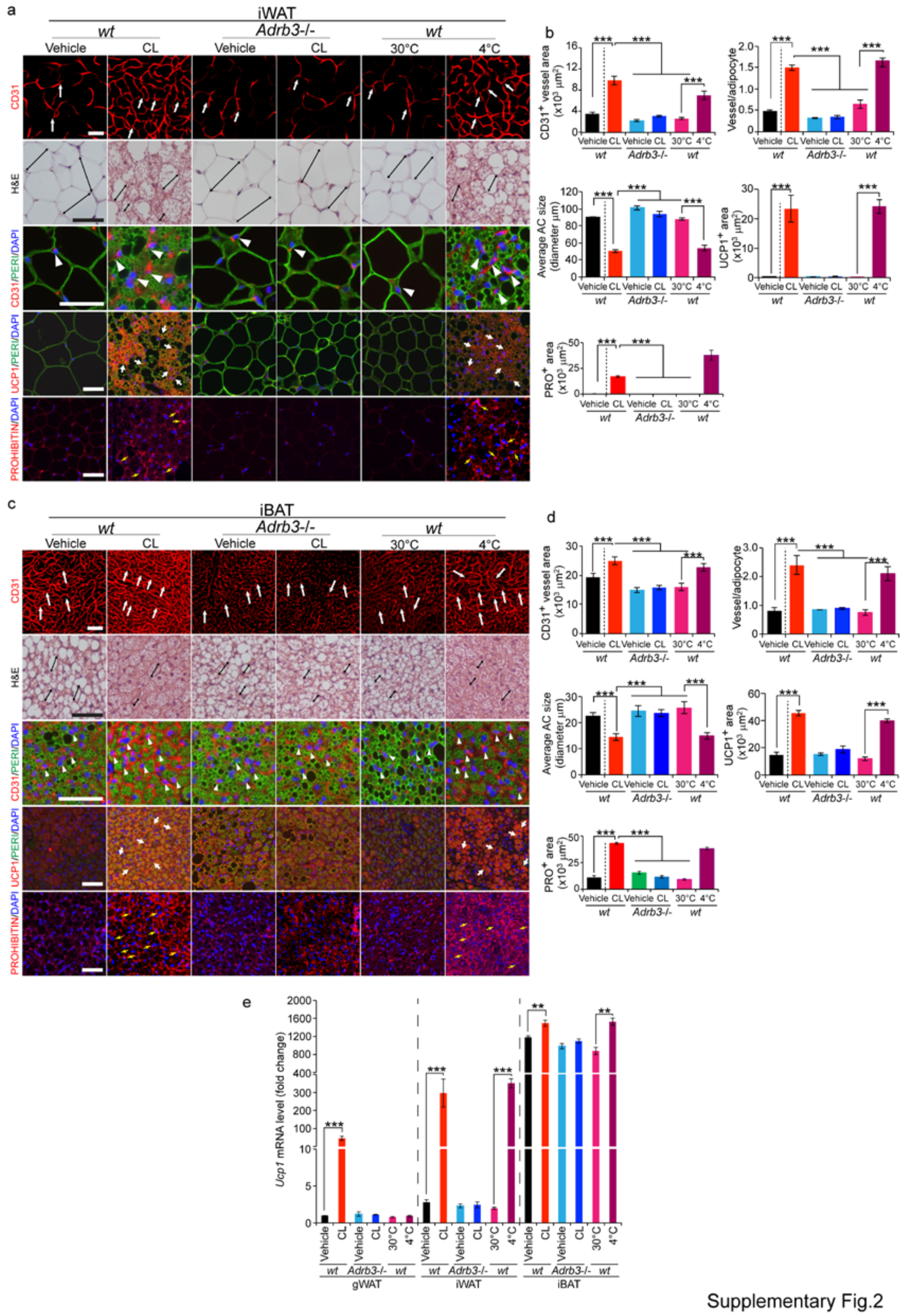

Supplementary Figure 2. β3-adrenoceptor-dependent activation of adipose angiogenesis and the beige phenotype in various adipose tissue.

(a) Microvessels ($CD31^+$ red) and adipocyte morphology (H&E; $Perilipin^+$ green), UCP1 expression ($UCP1^+$ red) and mitochondrial staining ($Prohibitin^+$ red) of vehicle- and CL-treated iWAT in *wt* and *Adrb3-/-* mice. Cold and thermoneutral temperature-exposed iWAT served as controls. White arrows and arrowheads in upper 3 panels point to microvessels. Arrows in second lowest row of panels indicate UCP1 positive signals. Yellow arrows point to prohibitin positive signals. n = 5 mice for each group.

(b) Quantification of microvessel density (n = 10 random fields; n = 5 mice for each group), average adipocyte size (> 30 adipocytes/field; n = 10 random fields; n = 5 mice for each group), UCP1 positive signals (n = 10 random fields; n = 5 mice for each group) and prohibitin positive signals (n = 10 random fields; n = 5 mice for each group) in CL- or temperature-treated groups (n = 5 mice for each group).

(c) Microvessels ($CD31^+$ red) and adipocyte morphology (H&E; $Perilipin^+$ green), UCP1 expression ($UCP1^+$ red) and mitochondrial staining ($Prohibitin^+$ red) of vehicle and CL-treated iBAT in *wt* and *Adrb3-/-* mice. Cold and thermoneutral temperature-exposed iBAT served as controls. White arrows and arrowheads in upper 3 panels point to microvessels. Arrows in second lowest row of panels indicate UCP1 positive signals. Yellow arrows point to prohibitin positive signals. n = 5 mice for each group.

(d) Quantification of microvessel density (n = 10 random fields; n = 5 mice for each group), average adipocyte size (> 30 adipocytes/field; n = 10 random fields; n = 5 mice for each group), UCP1 positive signals (n = 10 random fields; n = 5 mice for each group) and prohibitin positive signals (n = 10

random fields; n = 5 mice for each group) in various agent- or temperature-treated groups (n = 5 mice for each group).

(e) Comparison of *Ucp1* mRNA expression levels in gWAT, iWAT, and iBAT of vehicle- or CL-treated *wt* and *Adrb3-/-* mice and of 4°C- or 30°C-exposed *wt* mice (n = 10 samples for each group). These are standardized by *wt* vehicle-treated gWAT.

All scale bars, 50 μm. $^{**}p < 0.01$; $^{***}p < 0.001$ by two-sided unpaired *t*-test. Data presented as mean ± s.e.m.

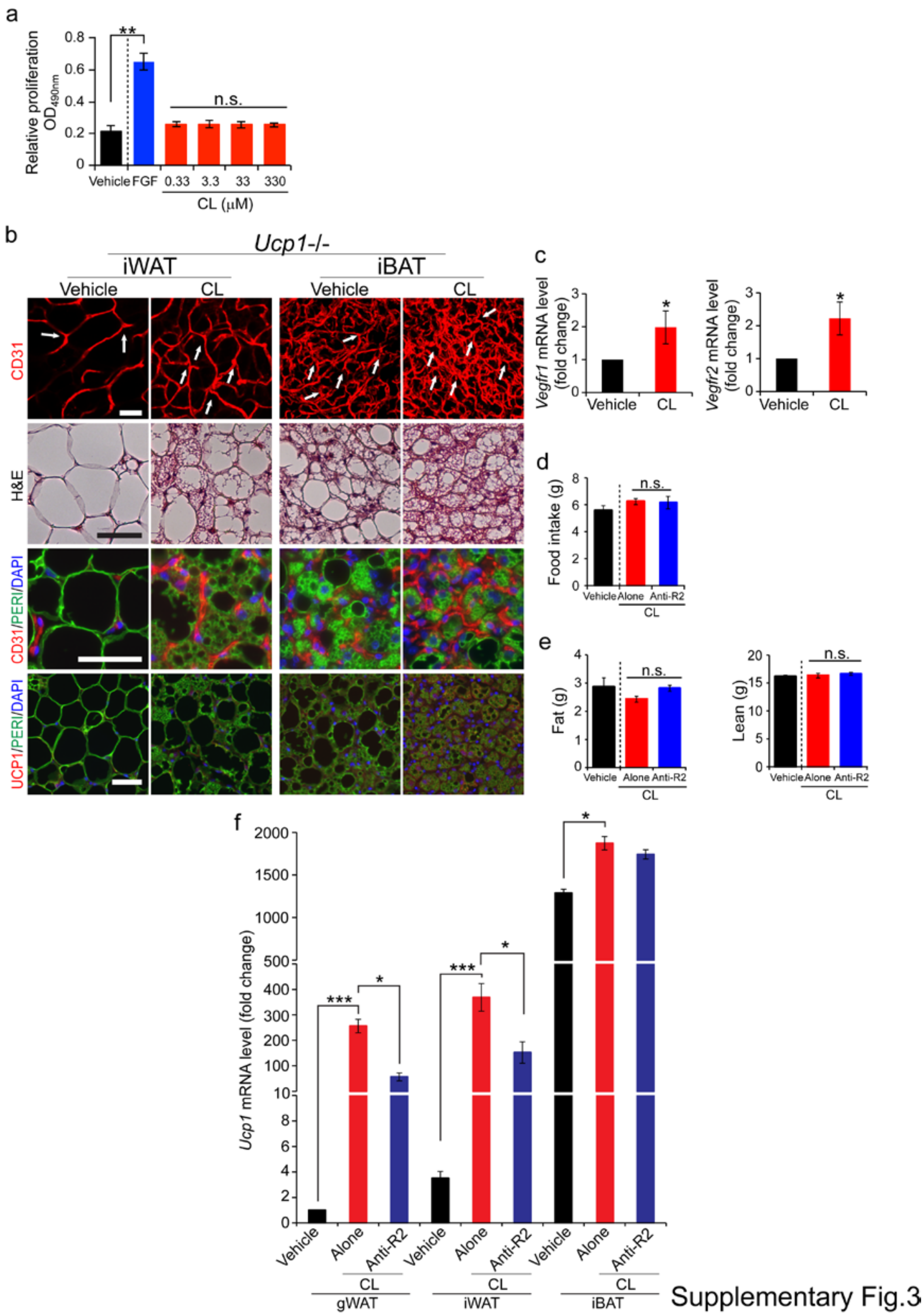

Supplementary Figure 3. EC proliferation assay, CL-induced UCP1-independent adipose angiogenesis in various adipose depots, effect of VEGF and VEGFR blockades.

(a) Assessment of CL-treated and non-treated EC proliferation at 72 h. FGF-2 (FGF) at a concentration of 5 ng/ml served as a positive control. n.s. = not significant.

(b) Microvessels ($CD31^+$ red), adipocyte morphology (H&E; $Perilipin^+$ green), and UCP1 expression ($UCP1^+$ red) of vehicle and CL-treated iWAT and iBAT in *Ucp1-/-* mice (n = 8 mice for each group). White arrows point to microvessels.

(c) *Vegfr1* and *Vegfr2* mRNA expression levels in vehicle- and CL-treated total iWAT samples in *wt* mice (n = 10 samples for each group; n = 8 mice for each group).

(d) Quantification of food intake in vehicle- and CL-treated *wt* mice that received treatment with various anti-VEGF agents (n = 8 mice for each group). n.s. = not significant.

(e) Measurement and quantification of fat and lean mass by a magnetic resonance imaging (MRI) in vehicle- and CL-treated *wt* mice that received treatment with various anti-VEGF agents (n = 8 mice for each group). n.s. = not significant.

(f) Comparison of *Ucp1* mRNA expression levels in gWAT, iWAT, and iBAT of in vehicle- and CL-treated *wt* mice that received treatment with various anti-VEGF agents (n = 8 samples for each group). These are standardized by vehicle treated gWAT.

All scale bars, 50 μm. *$p < 0.05$; **$p < 0.01$ by two-sided unpaired *t*-test. Data presented as mean ± s.e.m.

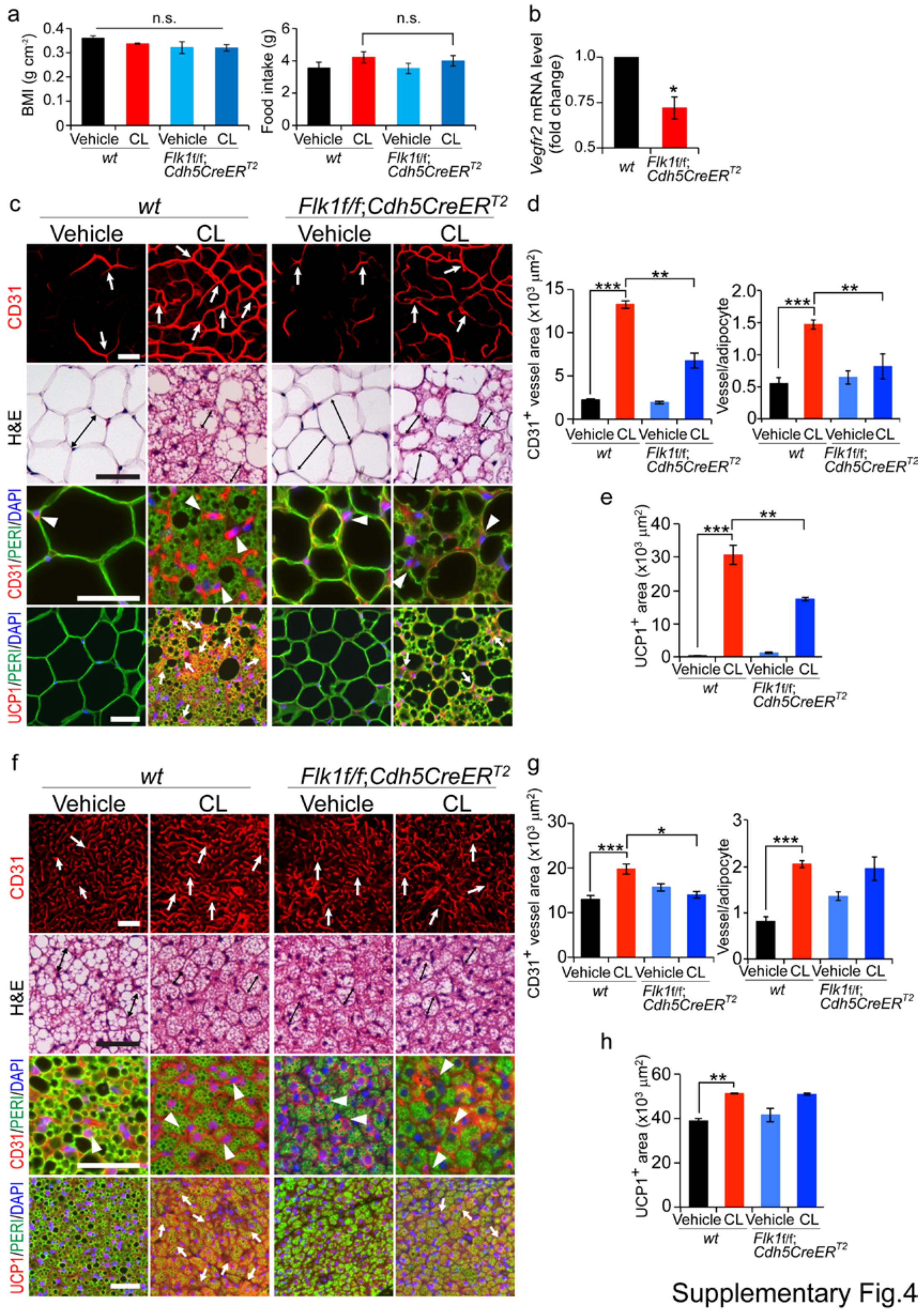

Supplementary Fig.4

Supplementary Figure 4. Body mass index, food intake, iWAT and iBAT angiogenesis defects, and iWAT-beige transition impairment in *Flk1f/f;Cdh5CreER^{T2}* mice.

(a) Quantification of body mass index (BMI) and food intake in vehicle- and CL-treated *wt* and *Flk1f/f;Cdh5CreER^{T2}* mice (n = 5 mice for each group). n.s. = not significant.

(b) *Vegfr2* mRNA expression levels in CD31^{+}EC from gWAT samples in each *wt* mice (n = 10 samples for each group) and *Flk1f/f;Cdh5CreER^{T2}* mice.

(c) Microvessels (CD31^{+} red), adipocyte morphology (H&E; Perilipin+ green), and UCP1 expression (UCP1+ red) of vehicle- and CL-treated iWAT in *wt* and *Flk1f/f;Cdh5CreER^{T2}* mice. White arrows point to microvessels. Arrowheads indicate UCP1 positive signals. n = 5 mice for each *wt* and *Flk1f/f;Cdh5CreER^{T2}* group.

(d) Quantification of iWAT microvessel density in vehicle- and CL-treated *wt* and *Flk1f/f;Cdh5CreER^{T2}* mice (n = 10 random fields; n = 5 mice for each group).

(e) Quantification of UCP1 positive staining signals (n = 10 random fields; n = 5 mice for each group).

(f) Microvessels (CD31^{+} red), adipocyte morphology (H&E; Perilipin^{+} green) and UCP1 expression (UCP1^{+} red) of vehicle- and CL-treated iBAT in *wt* and *Flk1f/f;Cdh5CreER^{T2}* mice (n = 10 random fields; n = 5 mice for each group). White arrows point to microvessels. Arrowheads indicate UCP1 positive signals.

(g) Quantification of iBAT microvessel density in vehicle- and CL-treated *wt* and *Flk1f/f;Cdh5CreER^{T2}* mice (n = 10 random fields; n = 5 mice for each group).

(h) Quantification of UCP1 positive staining signals (n = 10 random fields; n = 5 mice for each group).

All scale bars, 50 μm. $*p < 0.05$; $**p < 0.01$; $***p < 0.001$ by two-sided unpaired *t*-test. Data presented as mean ± s.e.m.

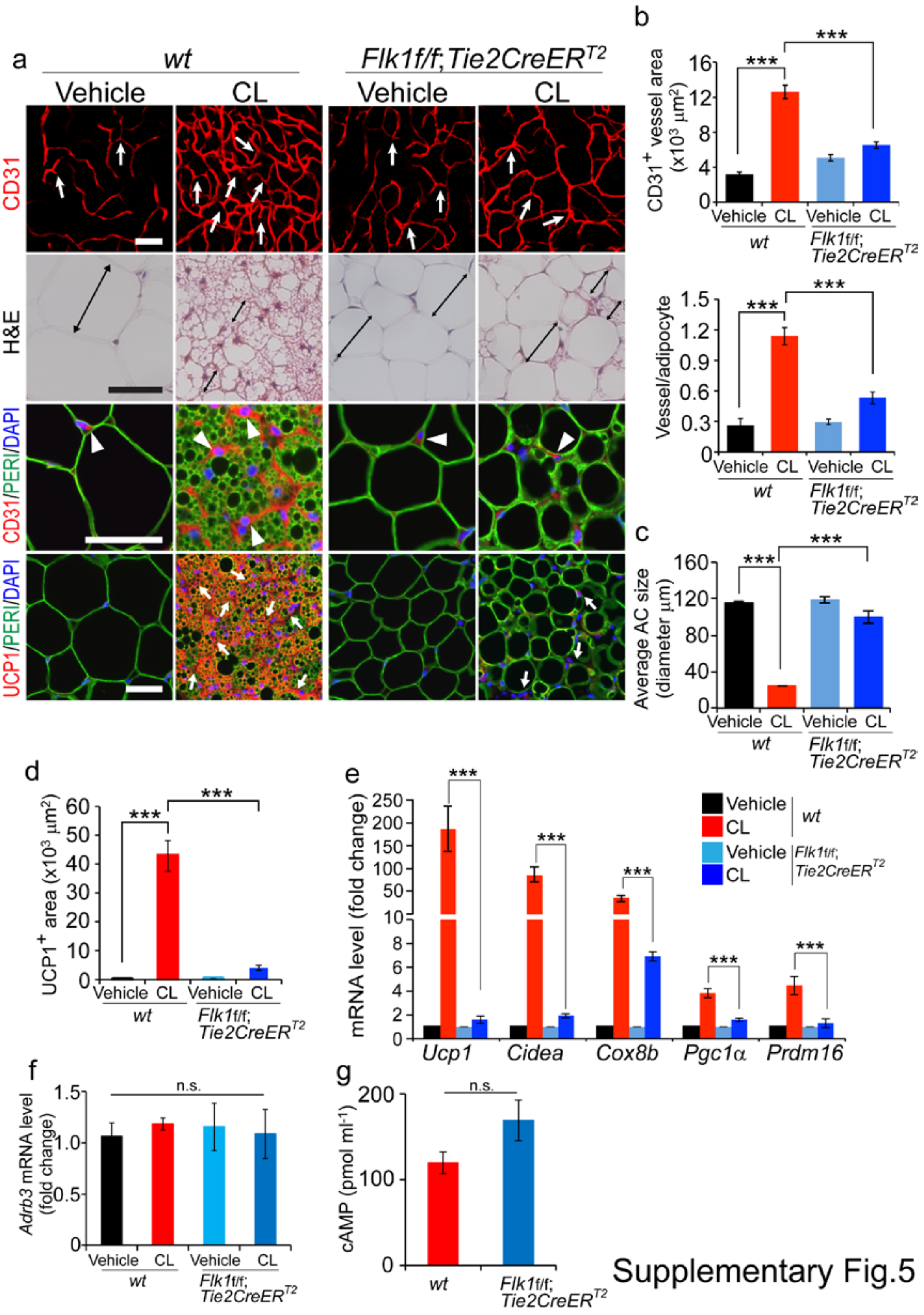

Supplementary Figure 5. Impaired CL-induced angiogenesis and beige transition in *Flk1f/f;Tie2CreER*^{T2} mice.

(a) Microvessels ($CD31^+$ red), adipocyte morphology (H&E; $Perilipin^+$ green), and UCP1 expression ($UCP1^+$ red) of vehicle- and CL-treated gWAT in *wt* and *Flk1f/f;Tie2CreER*^{T2} mice. White arrows and arrowheads in upper 3 panels point to microvessels. Arrows in the lowest row of panels indicate UCP1 positive signals. Double arrowed bars mark adipocyte diameter. n = 5 mice for each *wt* and *Flk1f/f;Tie2CreER*^{T2} group.

(b) Quantification of microvessel density in vehicle- and CL-treated *wt* and *Flk1f/f;Tie2CreER*^{T2} mice (n = 10 random fields; n = 5 mice for each group).

(c) Quantification of average adipocyte size (> 30 adipocytes/field; n = 10 random fields; n = 5 mice for each group).

(d) Quantification of UCP1 positive signals (n = 10 random fields; n = 5 mice for each group).

(e) qPCR quantification of mRNA expression levels *Ucp1, Cidea, Cox8b, Pgc1*α*,* and *Prdm16* in vehicle- and CL-treated total gWAT samples in *wt* and *Flk1f/f;Tie2CreER*^{T2} mice. (n = 10 samples for each group).

(f) qPCR quantification of mRNA expression levels *Adrb3* in vehicle- and CL-treated total gWAT samples in *wt* and *Flk1f/f;Tie2CreER*^{T2} mice. (n = 10 samples for each group). n.s. = not significant. cAMP levels in CL-treated total gWAT samples in *wt* and *Flk1f/f;Tie2CreER*^{T2} mice. (n = 5 samples for each group). n.s. = not significant. All scale bars, 50 μm. *p < 0.05; **p < 0.01; ***p < 0.001 by two-sided unpaired *t*-test. Data presented as mean ± s.e.m.

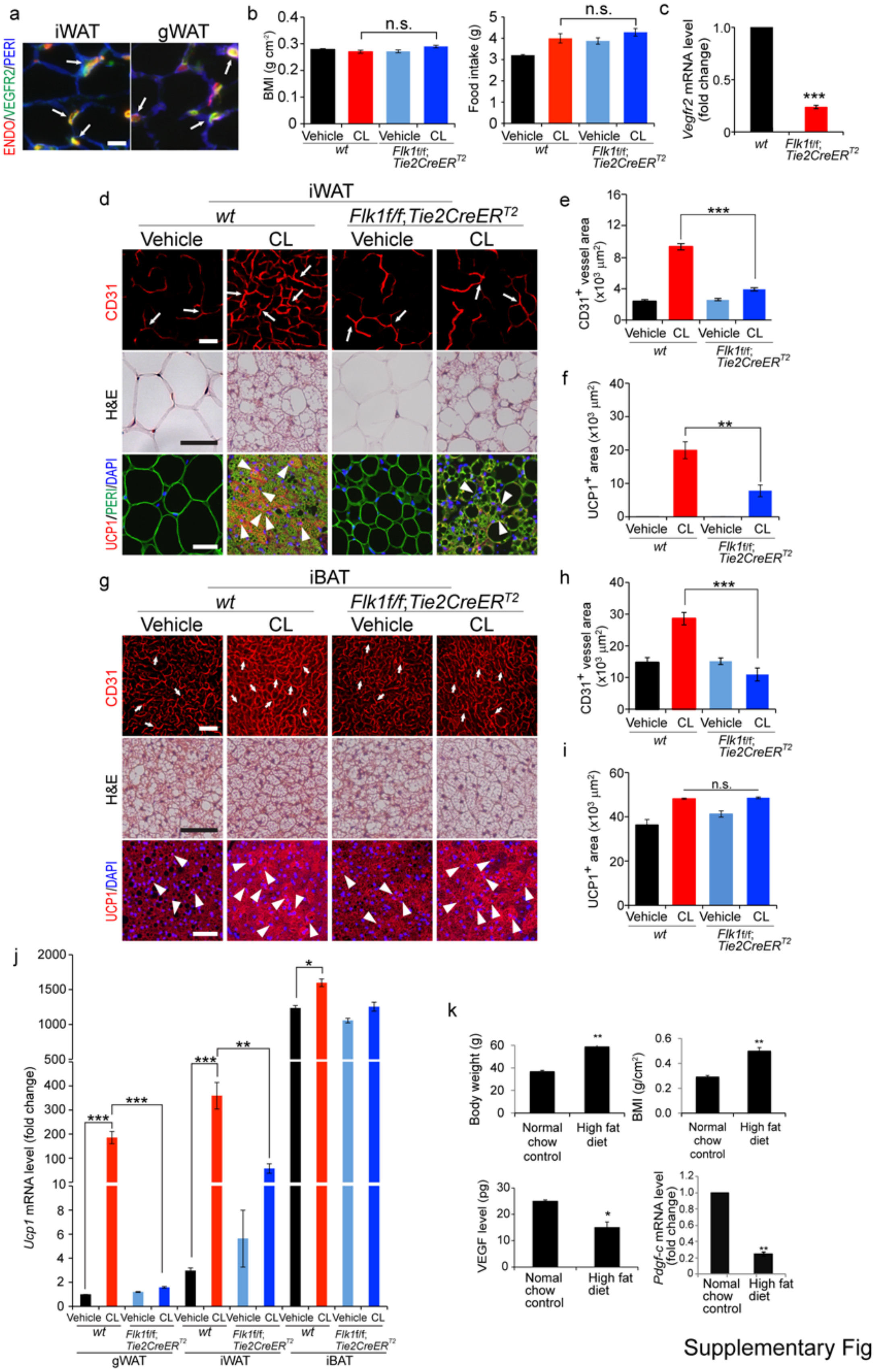

Supplementary Fig.6

Supplementary Figure 6. BMI, food intake, iWAT and iBAT angiogenesis defects, and iWAT-beige transition impairment in *Flk1f/f;Tie2CreER^{T2}* mice.

(a) Colocalization of VEGFR2 with endomucin in iWAT and gWAT (arrows point to overlapping signals. n = 5 mice for each group.

(b) Quantification of BMI and food intake in vehicle- and CL-treated *wt* and *Flk1f/f;Tie2CreER^{T2}* mice (n = 5 mice for each group). n.s. = not significant.

(c) *Vegfr2* mRNA expression levels in CD31^{+}EC from gWAT samples in each *wt* mice (n = 10 samples for each group) and *Flk1f/f;Tie2CreER^{T2}* mice.

(d) Microvessels (CD31^{+} red), adipocyte morphology (H&E; Perilipin^{+} green), and UCP1 expression (UCP1^{+} red) of vehicle- and CL-treated iWAT in *wt* and *Flk1f/f;Tie2CreER^{T2}* mice (n = 5 mice for each group). White arrows point to microvessels. Arrowheads indicate UCP1 positive signals.

(e) Quantification of iWAT microvessel density in vehicle- and CL-treated *wt* and *Flk1f/f;Tie2CreER^{T2}* mice (n = 10 random fields; n = 5 mice for each group).

(f) Quantification of UCP1 positive staining signals (n = 10 random fields; n = 5 mice for each group).

(g) Microvessels (CD31^{+} red), adipocyte morphology (H&E; Perilipin^{+} green) and UCP1 expression (UCP1^{+} red) of vehicle- and CL-treated iBAT in *wt* and *Flk1f/f;Tie2CreER^{T2}* mice. White arrows point to microvessels. Arrowheads indicate UCP1 positive signals. n = 5 mice for each group.

(h) Quantification of iBAT microvessel density in vehicle- and CL-treated *wt* and *Flk1f/f;Tie2CreER^{T2}* mice (n = 10 random fields; n = 5 mice for each group).

(i) Quantification of UCP1 positive staining signals (n = 10 random fields; n = 5 mice for each group). n.s. = not significant.

(j) Comparison of *Ucp1* mRNA expression levels in gWAT, iWAT, and iBAT of in vehicle- and CL-treated *wt* mice and *Flk1f/f;Tie2CreER*^{T2} mice (n = 10 samples for each group). These are standardized by *wt* vehicle treated gWAT.

(k) Body weight and BMI of HFD-fed obese mice versus healthy mice (n = 4 mice for each group). Expression levels of VEGF and PDGF-C in iWAT of HFD-fed obese and healthy mice (n = 8 samples for each group).

All scale bars, 50 μm. *$p < 0.05$; **$p < 0.01$; ***$p < 0.001$ by two-sided unpaired *t*-test. Data presented as mean ± s.e.m.

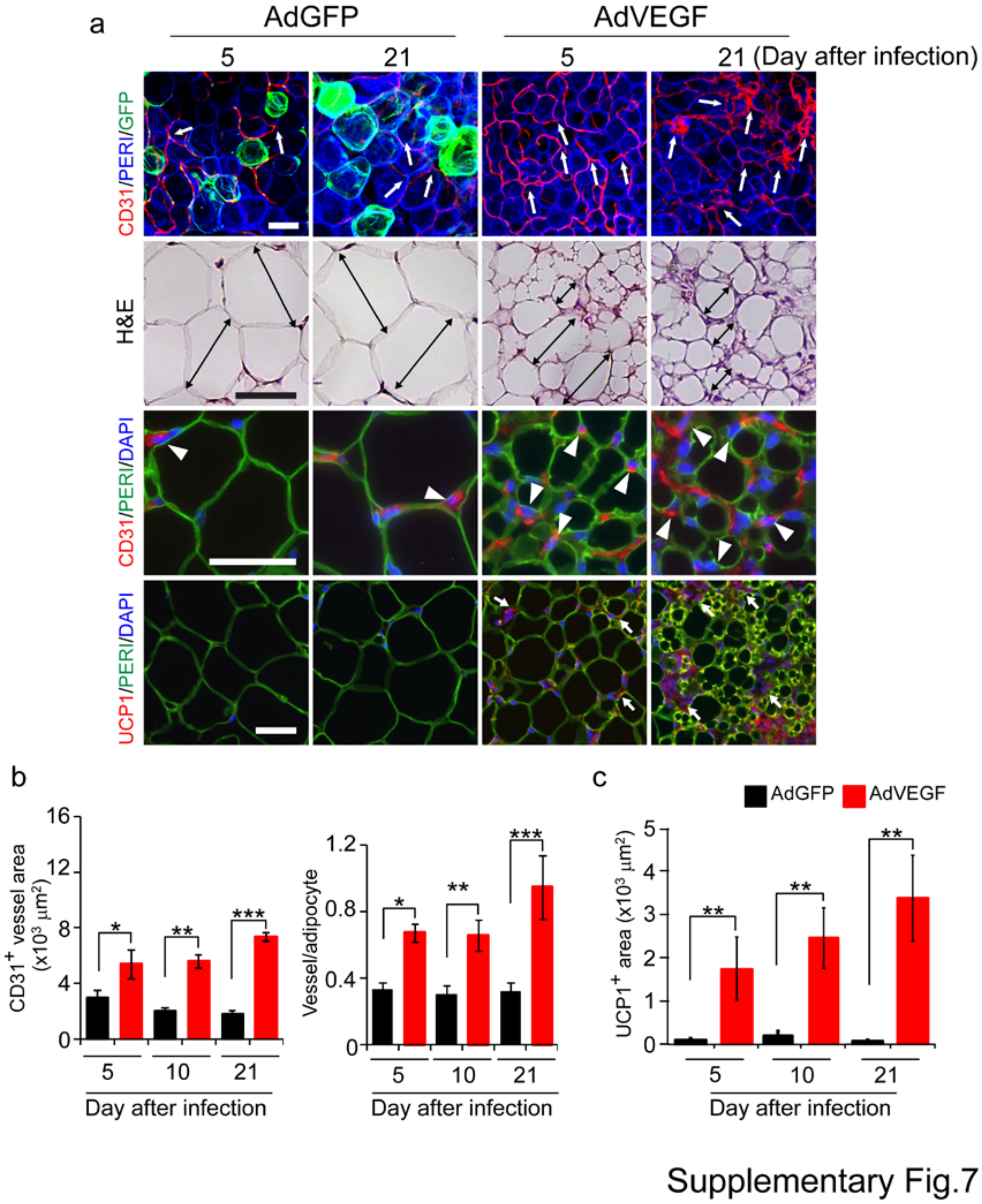

Supplementary Figure 7. Time course effects of AdVEGF delivery on adipose angiogenesis and beige transition.

(a) Microvessels (CD31$^+$ red), adipocyte morphology (H&E; Perilipin$^+$ green), and UCP1 expression (UCP1$^+$ red) of gWAT in mice that were treated with AdGFP and AdVEGF. White arrows and arrowheads in upper 3 panels point to microvessels. Double arrowed bars mark adipocyte diameter. Arrows in the lowest row of panels indicate UCP1 positive signals. Green in the top

row of panels indicates GFP positive signals. n = 5 mice for NAT group, n = 10 mice for each AdGFP and AdVEGF group.

(b) Quantification of microvessel density in AdGFP- and AdVEGF-treated (n = 10 random fields; n = 5 mice for NAT group, n = 10 mice for each AdGFP and AdVEGF group).

(c) Quantification of UCP1 positive signals (n = 10 random fields; n = 5 mice for NAT group, n = 10 mice for each AdGFP and AdVEGF group).

All scale bars, 50 μm. *$p < 0.05$; **$p < 0.01$; ***$p < 0.001$ by two-sided unpaired *t*-test. Data presented as mean ± s.e.m.

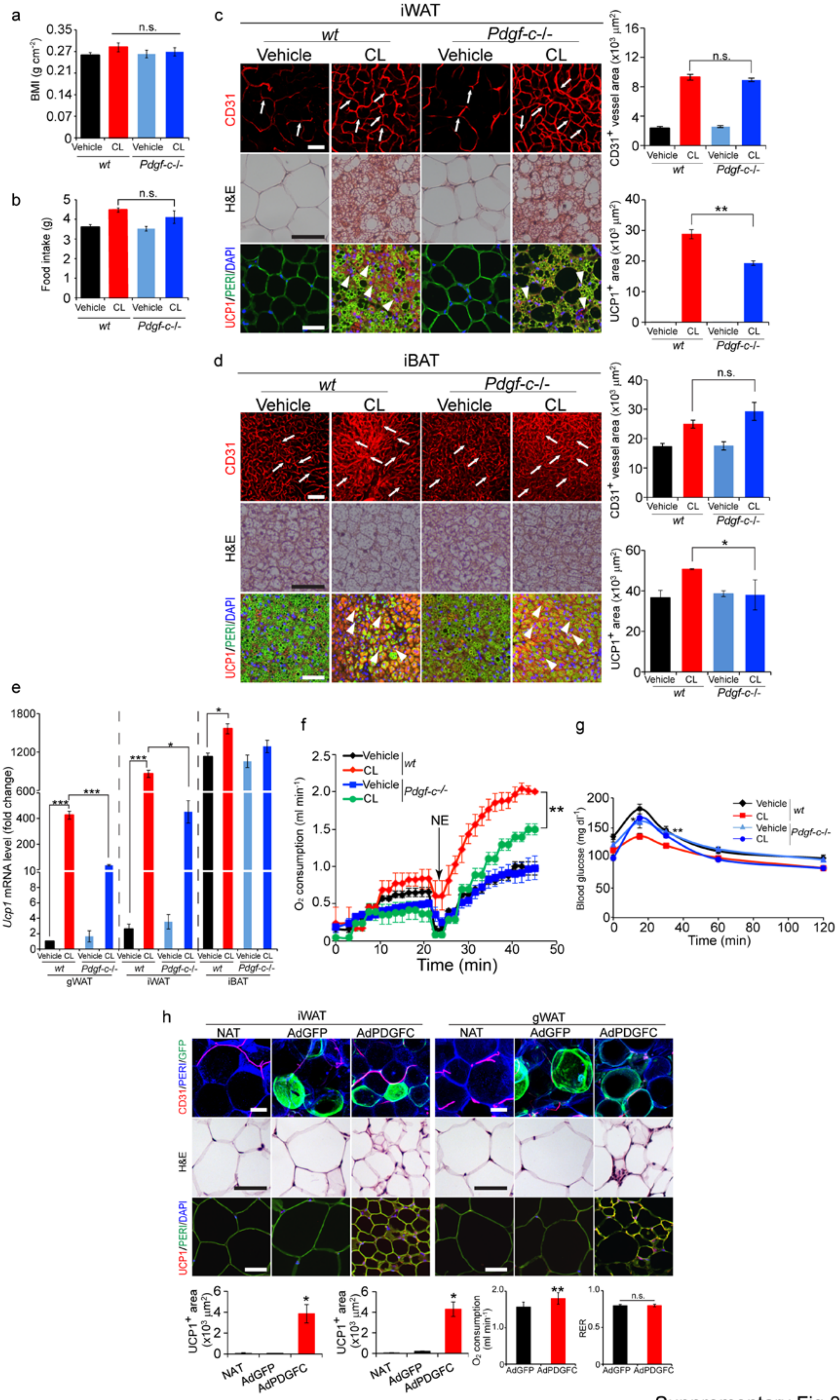

Supprementary Fig.8

Supplementary Figure 8. BMI, food intake, iWAT-beige transition impairment, thermogenic metabolism, glucose tolerance test in *Pdgf-c-/-* mice, and gain-of-function by delivery of AdPDGFC into adipose in obese mice.

(a) Quantification of BMI in vehicle- and CL-treated *wt* and *Pdgf-c-/-* mice (n = 5 mice for each group). n.s. = not significant.

(b) Quantification of food intake in vehicle- and CL-treated *wt* and *Pdgf-c-/-* mice (n = 5 mice for each group). n.s. = not significant.

(c) Microvessels ($CD31^+$ red), adipocyte morphology (H&E; $Perilipin^+$ green), and UCP1 expression ($UCP1^+$ red) of vehicle- and CL-treated iWAT in *wt* and *Pdgf-c-/-* mice. White arrows point to microvessels. Arrowheads indicate UCP1 positive signals. Quantification of microvessel density (n = 10 random fields; n = 5 mice for each group) and UCP1 positive signals (n = 10 random fields; n = 5 mice for each group) in vehicle- and CL-treated *wt* and *Pdgf-c-/-* mice (n = 5 mice for each group). n.s. = not significant.

(d) Microvessels ($CD31^+$ red), adipocyte morphology (H&E; $Perilipin^+$ green), and UCP1 expression ($UCP1^+$ red) of vehicle- and CL-treated iBAT in *wt* and *Pdgf-c-/-* mice. White arrows point to microvessels. Arrowheads indicate UCP1 positive signals. Quantification of microvessel density (n = 10 random fields; n = 5 mice for each group) and UCP1 positive signals (n = 10 random fields; n = 5 mice for each group) in vehicle- and CL-treated *wt* and *Pdgf-c-/-* mice (n = 5 mice for each group). n.s. = not significant.

(e) Comparison of *Ucp1* mRNA expression levels in gWAT, iWAT, and iBAT of in vehicle- and CL-treated *wt* mice and *Pdgf-c-/-* mice (n = 12 samples for each group). These are standardized by *wt* vehicle treated gWAT.

(f) Norepinephrine-stimulated non-shivering thermogenesis in vehicle- or CL-treated *wt* and *Pdgf-c-/-* (n = 5 mice for each group). NE = norepinephrine.

(g) Oral glucose tolerance in vehicle- or CL-treated *wt* and *Pdgf-c-/-* (n = 8 mice for each group).

(h) Microvessels ($CD31^+$ red), adipocyte morphology (H&E; $Perilipin^+$ blue in top rows of panels and green in bottom rows of panels), and UCP1 expression ($UCP1^+$ red) of gWAT in mice that were treated with AdGFP and AdPDGFC (green in top rows of panels). Green in the top row of panels indicates GFP positive signals. Quantification of UCP1 positive signals (n = 10 random fields/group; n = 6 mice for each group) and energy expenditures (n = 4 mice for each group).

All scale bars, 50 μm. *p < 0.05; **p < 0.01; ***p < 0.001 by two-sided unpaired *t*-test. Data presented as mean ± s.e.m.

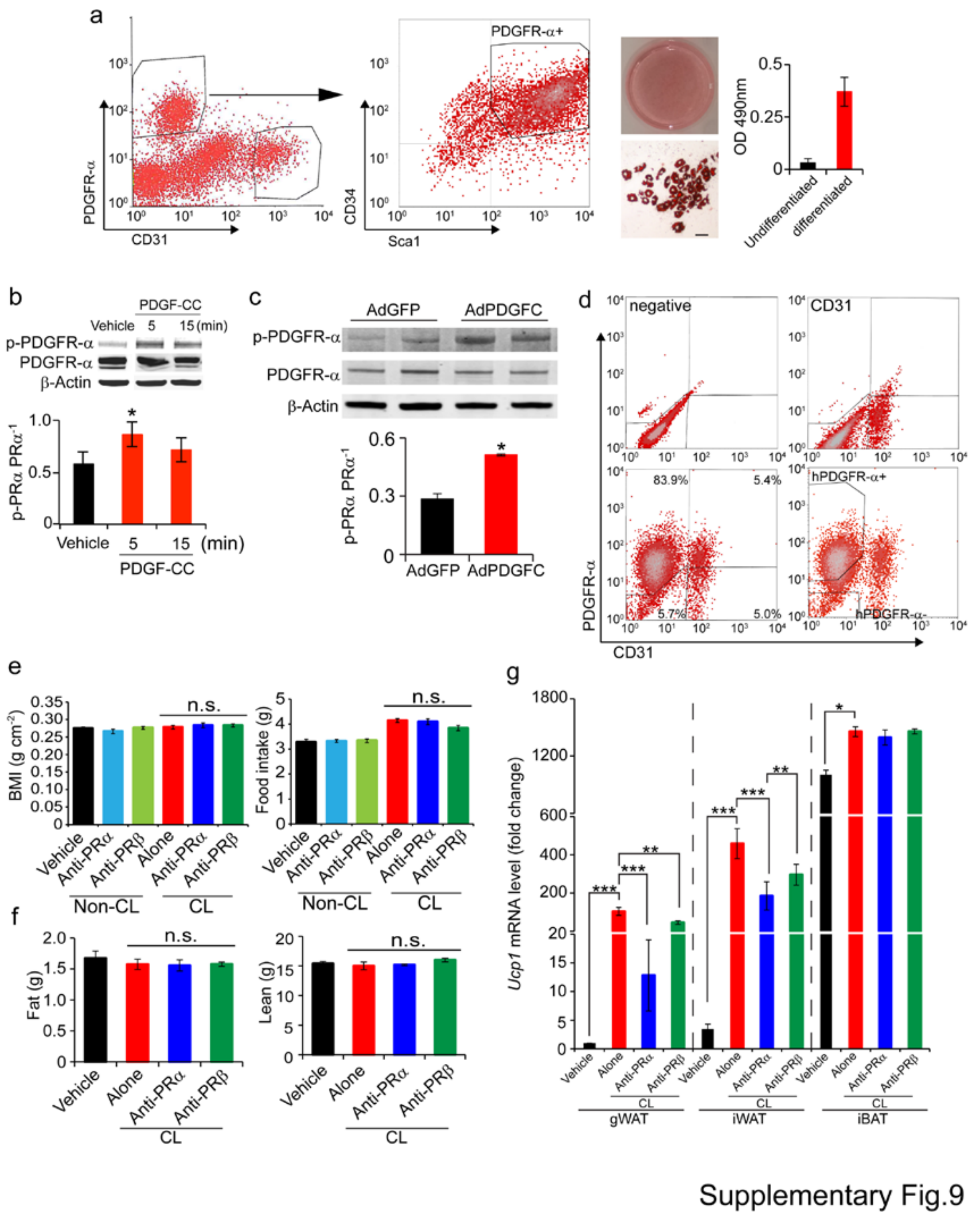

Supplementary Figure 9. FACS sorting of mouse and human PDGFR-α$^+$ Cells, *in vitro* assay, and PDGFR blockades CL-induced UCP1 expression.

(a) FACS analysis and isolation of the CD31$^-$, PDGFR-α$^+$ and Sca1$^+$ expressing cell fraction in SVF isolated from gWAT in *wt* mice. Oil red O staining and the quantification.

(b) Activation of PDGFR-α by PDGF-CC (100 ng/ml) in differentiated PDGFR-α^+ cells isolated from gWAT.

(c) Activation of PDGFR-α by delivery of AdPDGFC into WAT.

(d) FACS analysis and isolation of the $CD31^-$ and PDGFR-α^+ expressing cell fraction in SVF isolated from subcutaneous WAT in human patient.

(e) Quantification of BMI and food intake in vehicle- and CL-treated *wt* mice that received PDGFR-α (PRα) blockade and PDGFR-β (PRβ) blockade treatment (n = 8 mice for each group). n.s. = not significant.

(f) Quantification of fat and lean mass of the mice measured by MRI in vehicle- and CL-treated *wt* mice that received treatment with received PRα- and PRβ- blockade treatment (n = 8 mice for each group). n.s. = not significant.

(g) Comparison of *Ucp1* mRNA expression levels in gWAT, iWAT, and iBAT of in vehicle- and CL-treated *wt* mice that received PRα- and PRβ-blockade treatment (n = 16 samples for each group). These are standardized by *wt* vehicle treated gWAT.

All scale bars, 50 μm. *$p < 0.05$; **$p < 0.01$; ***$p < 0.001$ by two-sided unpaired *t*-test. Data presented as mean ± s.e.m.

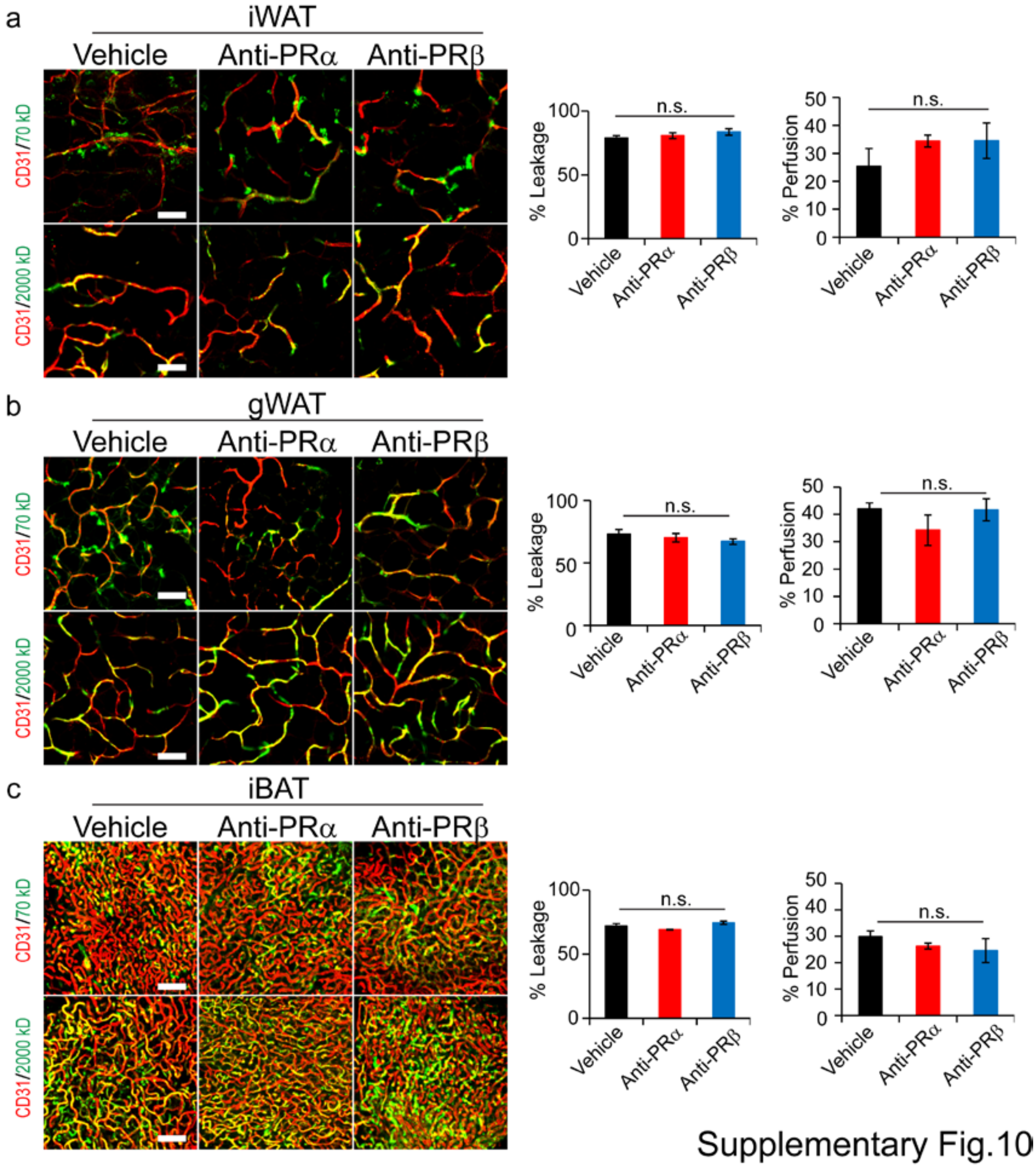

Supplementary Figure 10. Blood perfusion and leakiness of PDGFR-α- and PDGFR-β- blockades treated iWAT, gWAT and iBAT.

(a-c) CD31^{+} microvessel density, vascular permeability of 70 kD dextran, and perfusion of 2000 kD dextran in iWAT, gWAT and iBAT. Positive signals in each measured parameters were quantified from 10 random fields/group (n = 5 mice for each group). n.s. = not significant. All scale bars, 50 μm. Data presented as mean ± s.e.m.

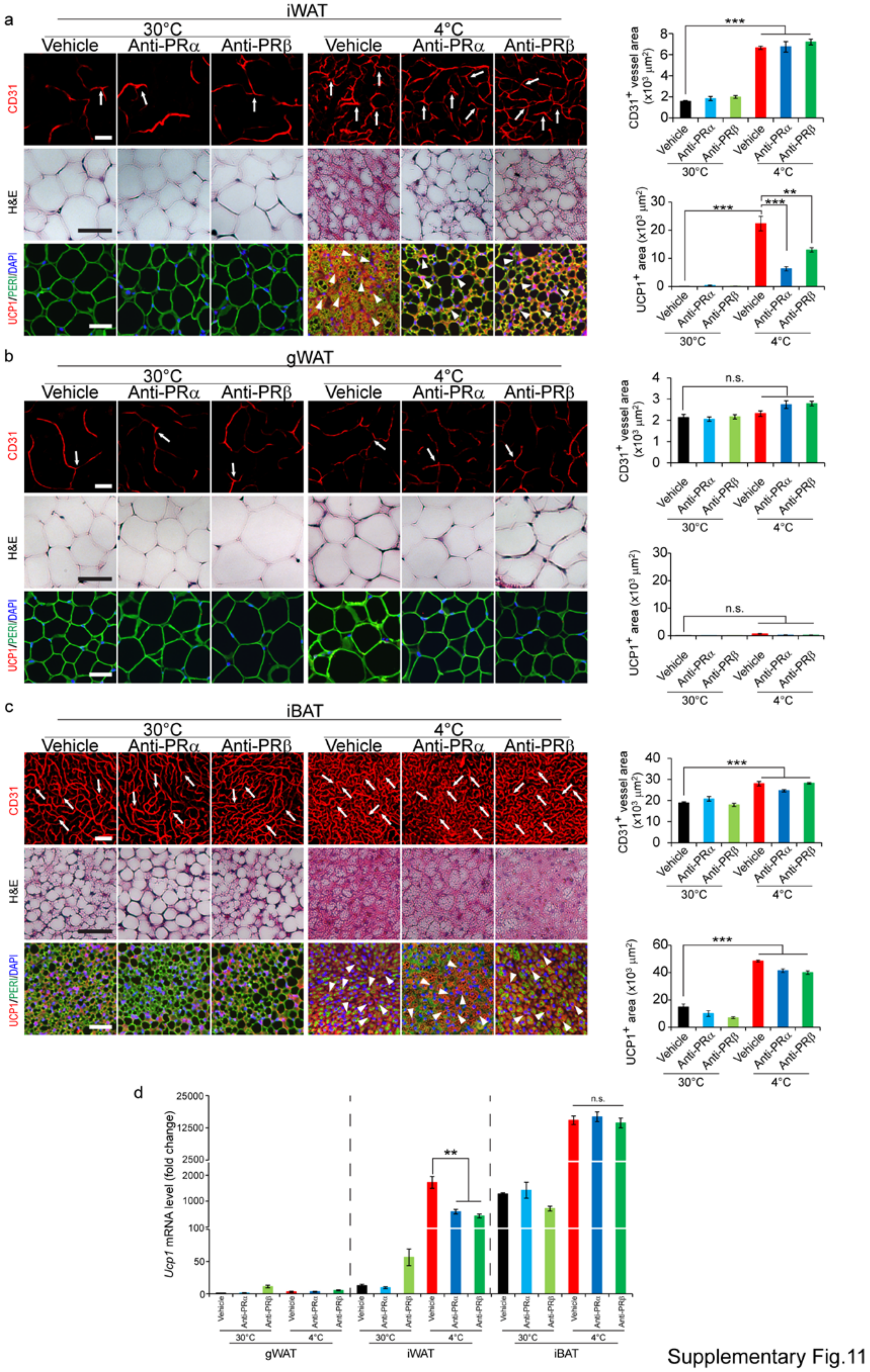

Supplementary Fig.11

Supplementary Figure 11. Effect of PDGFR blockades on cold exposure induced adipose angiogenesis and the beige phenotype in various adipose tissue.

(a) Microvessels ($CD31^+$ red), adipocyte morphology (H&E; $Perilipin^+$ green), and UCP1 expression ($UCP1^+$ red) of vehicle- and PDGFR blockades-treated iWAT in 4°C- or 30°C- exposed *wt* mice that received PRα- and PRβ- blockade treatment (n = 8 mice for each group). White arrows point to microvessels. Arrowheads indicate UCP1 positive signals. Quantification of microvessel density (n = 10 random fields; n = 8 mice for each group) and UCP1 positive signals (n = 10 random fields; n = 8 mice for each group). n.s. = not significant.

(b) Microvessels ($CD31^+$ red), adipocyte morphology (H&E; $Perilipin^+$ green), and UCP1 expression ($UCP1^+$ red) of vehicle- and PDGFR blockades-treated gWAT in 4°C- or 30°C- exposed *wt* mice that received PRα- and PRβ- blockade treatment (n = 8 mice for each group). White arrows point to microvessels. Arrowheads indicate UCP1 positive signals. Quantification of microvessel density (n = 10 random fields; n = 8 mice for each group) and UCP1 positive signals (n = 10 random fields; n = 8 mice for each group). n.s. = not significant.

(c) Microvessels ($CD31^+$ red), adipocyte morphology (H&E; $Perilipin^+$ green), and UCP1 expression ($UCP1^+$ red) of vehicle- and PDGFR blockades-treated iBAT in 4°C- or 30°C- exposed *wt* mice that received PRα- and PRβ- blockade treatment (n = 8 mice for each group). White arrows point to microvessels. Arrowheads indicate UCP1 positive signals. Quantification of microvessel density (n = 10 random fields; n = 8 mice for each group) and UCP1 positive signals (n = 10 random fields; n = 8 mice for each group).

(d) Comparison of *Ucp1* mRNA expression levels in gWAT, iWAT, and iBAT of in 4°C- or 30°C- exposed *wt* mice that received PRα- and PRβ-blockade treatment (n = 12 samples; n = 8 mice for each group). These are standardized by *wt* 30°C- exposed gWAT. n.s. = not significant.

All scale bars, 50 μm. $^{**}p < 0.01$; $^{***}p < 0.001$ by two-sided unpaired *t*-test. Data presented as mean ± s.e.m.

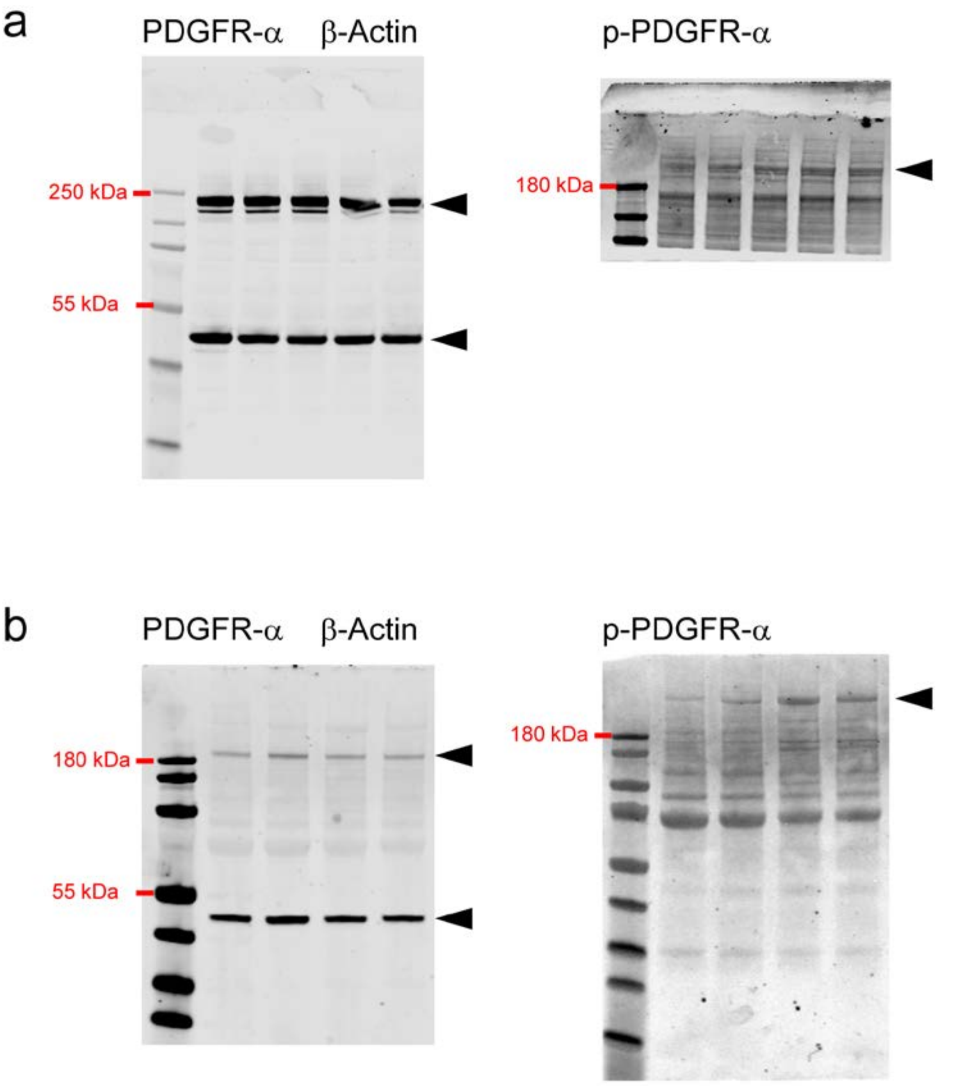

Supplementary Fig.12

Supplementary Fig. 12. Original full-length western blots used for Supplementary Figures 9

(a) Original western blots used for Supplementary Fig. 9b.

(b) Original western blots used for Supplementary Fig. 9c.

Supplementary Table 1 Nucleotide Sequences of Primers Used for qPCR

Gene	**Species**	**Forward primer sequence**	**Reverse primer sequence**
Vegf	mouse	5'-ATCCGCATGATCTGCATGG-3'	5'-AGTCCCATGAAGTGATCAAGTTCA-3'
Vegfr1	mouse	5'-AGCCCACCTCTCTATCCGCTGG-3'	5'-GGCGCTTCCGAATCTCTAACG-3'
Vegfr2	mouse	5'-CTCTGTGGGTTTGCCTGGCGATTTTCT-3'	5'GGGGATCACCACAGTTTTGTTCTTGTT-3'
Ucp1	mouse	5'-AAACAGAAGGATTGCCGAAA-3'	5'-TGCATTCTGACCTTCACGAC-3'
Pdgf-a	mouse	5'-GAGGGATGGTACTGAATTTCGC-3'	5'-TGCAAACTGCAGGAATGGCT-3'
Pdgf-b	mouse	5'-ATGTGCCCTTCAGTCTGCTC-3'	5'-GAGACAGGTCTCCTGCCCTA-3'
Pdgf-c	mouse	5'-GCCCGAAGTTTCCTCATACA-3'	5'-ACACTTCCATCACTGGGCTC-3'
Pdgf-d	mouse	5'-CGAGGGACTGTGCAGTAGAAA-3'	5'-TTGATGGATGCTCTCTGCGG-3'
Pgc1α	mouse	5'-AGCCGTGACCACTGACAACGAG-3'	5'-GCTGCATGGTTCTGAGTGCTAAG-3'
Cidea	mouse	5'-TGCTCTTCTGTATCGCCCAGT-3'	5'-GCCGTGTTAAGGAATCTGCTG-3'
Cox8b	mouse	5'-GAACCATGAAGCCAACGACT-3'	5'-GCGAAGTTCACAGTGGTTCC-3'
Prdm16	mouse	5'-CAGCACGGTGAAGCCATTC-3'	5'-GCGTGCATCCGCTTGTG-3'
Adrb3	mouse	5'-GGGAACGACAGCGACTTCTT-3'	5'-GCCAGGACGATAACCGACAT-3'
Actin	mouse	5'-AGGCCCAGAGCAAGAGAGG-3'	5'-TACATGGCTGGGGTGTTGAA-3'
Tie2Creert		5'-CGAGTGATGAGGTTCGCAAG-3'	5'-TGAGTGAACGAACCTGGTCG-3'
Cdh5Creert		5'-GCCTGCATTACCGGTCGATGCAACGA-3'	5'-GTGGCAGATGGCGCGGCAACACCATT-3'
Flk1 flox		5'-CCACAGAACAACTCAGGGCTA-3'	5'-GGGAGCAAAGTCTCTGGAAA-3'
Pdgf-c	wild type	5'-AGCTGACATTTGATGAGAGAT-3'	5'-AGTAGGTGAAATAAGAGGTGAACA-3'
	mutant	5'-CTGATGTTCTCGTGACTCTGA-3'	5'-TAGCTAGTCGATACCGTCGA-3'
Ucp1	human	5'-GCAGGGAAAGAAACAGCACCT-3'	5'-CCCGTGTAGCGAGGTTTGAT-3'
Gapdh	human	5'-CATTTCCTGGTATGACAACGA-3'	5'-GTCTACATGGCAACTGTGAG-3'
